# Astrocyte-Derived Interleukin 11 Modulates Astrocyte–Microglia Crosstalk via Nuclear Factor-κB Signaling Pathway in Sepsis-Associated Encephalopathy

**DOI:** 10.34133/research.0598

**Published:** 2025-01-30

**Authors:** Dandan Zhu, Peng Wang, Xiyue Chen, Kaituo Wang, Yunsong Wu, Min Zhang, Jianhua Qin

**Affiliations:** ^1^ Division of Biotechnology, Dalian Institute of Chemical Physics, Chinese Academy of Sciences, Dalian 116023, China.; ^2^ University of Science and Technology of China, Hefei 230026, China.; ^3^ Suzhou Institute for Advanced Research, University of Science and Technology of China, Suzhou 215123, China.; ^4^ Beijing Institute for Stem Cell and Regenerative Medicine, Chinese Academy of Sciences, Beijing 100000, China.; ^5^ Department of Critical Care Medicine, The Second Hospital of Dalian Medical University, Dalian 116023, China.

## Abstract

Sepsis-associated encephalopathy (SAE) is a severe and frequent septic complication, characterized by neuronal damage as key pathological features. The astrocyte–microglia crosstalk in the central nervous system (CNS) plays important roles in various neurological diseases. However, how astrocytes interact with microglia to regulate neuronal injury in SAE is poorly defined. In this study, we aim to investigate the molecular basis of the astrocyte–microglia crosstalk underlying SAE pathogenesis and also to explore the new therapeutic strategies targeting this crosstalk in this devastating disease. We established a human astrocyte/microglia coculture system on a microfluidic device, which allows real-time and high-resolution recording of glial responses to inflammatory stimuli. Based on this microfluidic system, we can test the responses of astrocytes and microglia to lipopolysaccharide (LPS) treatment, and identify the molecular cues that mediate the astrocyte–microglia crosstalk underlying the pathological condition. In addition, the SAE mouse model was utilized to determine the state of glial cells and evaluate the therapeutic effect of drugs targeting the astrocyte–microglia crosstalk in vivo. Here, we found that activated astrocytes and microglia exhibited close spatial interaction in the SAE mouse model. Upon LPS exposure for astrocytes, we detected that more microglia migrated to the central astrocyte culture compartment on the microfluidic device, accompanied by M1 polarization and increased cell motility in microglia. Cytokine array analysis revealed that less interleukin 11 (IL11) was secreted by astrocytes following LPS treatment, which further promoted reprogramming of microglia to pro-inflammatory M1 phenotype via the nuclear factor-κB (NF-κB) signaling pathway. Intriguingly, we found that IL11 addition markedly rescued LPS-induced neuronal injuries on the microfluidic system and brain injury in the SAE mouse model. This study defines an unknown crosstalk of astrocyte–microglia mediated by IL11, which contributed to the neuropathogenesis of SAE, and suggested a potential therapeutic value of IL11 in the devastating disease.

## Introduction

Sepsis-associated encephalopathy (SAE) is a severe septic complication, characterized by serious neuronal injury [1]. The underlying pathological mechanism in SAE is complicated and thus remains unclear. Human brain is one of the most complicated organs in the body, with numerous types of cells and complex intercommunications between distinctive cell types. Among them, astrocytes and microglia are 2 vital cell types, which play important roles in both physiological condition and neurological diseases [2–5]. Previous studies reported that astrocytes and microglia are closely associated with the pathogenesis of SAE [6,7]; however, it is still unclear how the 2 cell types coordinate synergistically to contribute to the SAE progression.

Currently, studying the intercellular crosstalk in SAE is challenging, primarily due to the limitations of ideal models that can accurately probe the human-relevant responses to pathological stimuli. Despite of valuable contributions from in vitro cell cultures and murine models [8,9], current models show a limited ability to detect the transient intercellular crosstalk mediated by secreted factors. For animal models, the complexities of the physiological environment in vivo pose challenges for real-time tracking and monitoring of cell response. In vitro models, such as monolayer cell cultures treated with lipopolysaccharide (LPS) [10,11], have limited capacity to model the complicated physiological environment and the astrocyte–microglia interactions. Therefore, it is an urgent need to establish a biomimetic neuronal model that enables recapitulation of the physiological condition, and study the astrocyte–microglia intercommunication underlying SAE in a human-relevant manner.

Microfabrication and microfluidic technologies have offered unprecedented opportunities to recapitulate their key pathological microenvironment in CNS diseases [12]. Microfluidic chip technology has made it possible to study intercommunication between 2 or more cell types of interest and identify the specific mechanisms involved, especially the investigation of cell signaling and monitoring cell migration behavior in real time. Recently, several microfluidic chip models have been used to study cell–cell interactions in CNS disorders, including Alzheimer’s disease (AD) [13], neuroinflammation [14], cancer [15], and so on [16,17].

In this study, we innovatively constructed a human astrocyte/microglia coculture system using a microfluidic device to investigate crosstalk between astrocytes and microglia and its impact on neurons in SAE. The microfluidic device comprises a central chamber and 2 side chambers connected by microgrooves, enabling the culture of astrocytes and microglia separately. Microglial migration through the microgrooves and into the adjacent compartment is visible in real time. The microgrooves linking the 2 chambers possess sufficient resistance to maintain a hydrostatic pressure difference between the compartments, enabling the containment and isolation of a biomolecular insult (e.g., cytokines) in the lesser volume compartment. Based on this astrocyte/microglia coculture system, we detected more microglia that migrated to the central astrocyte culture compartment following LPS treatment in the astrocyte culture compartment, accompanied by M1 polarization and increased cell motility in microglia. Cytokine array analysis revealed that less interleukin 11 (IL11) was secreted by astrocytes following LPS treatment, which further promoted reprogramming of microglia to pro-inflammatory M1 phenotype via the nuclear factor-κB (NF-κB) signaling pathway. Intriguingly, we found that adding IL11 significantly reversed microglial activation and rescued LPS-induced neuronal injuries on the microfluidic system and brain injury in the SAE mouse model. In summary, this study identifies astroglia-derived IL11 as a key messenger for mediating astrocyte–microglia inter-crosstalk in the pathogenesis of SAE, and it might be utilized as a target of therapeutic intervention for this devastating disease.

## Results

### CLP mouse model revealed spatial interaction between astrocytes and microglia in the brain hippocampus

**Fig. 1.**
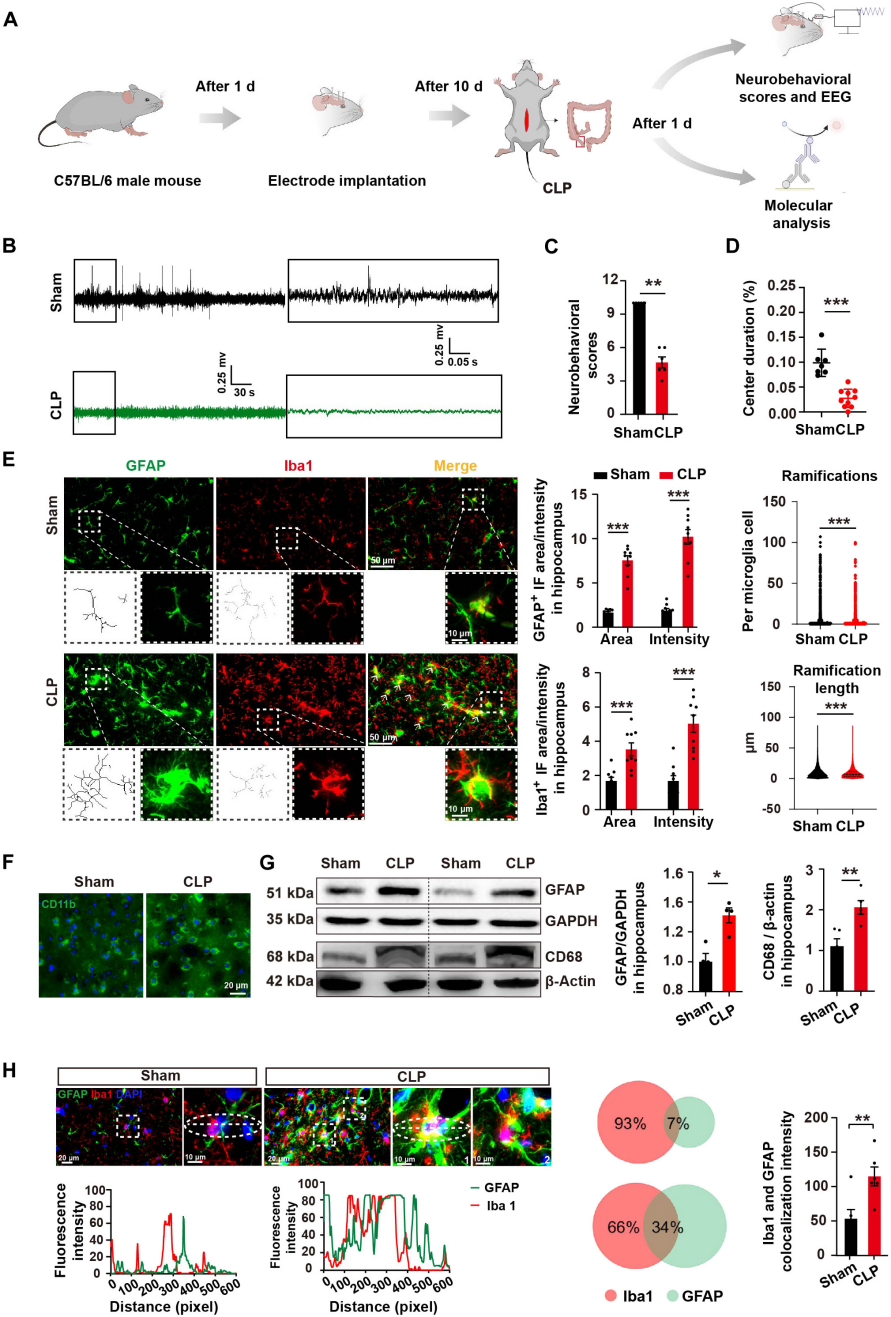
Interaction between astrocytes and microglia in the hippocampus of CLP mice. (A) Experimental timeline. (B) Representative electroencephalogram (EEG) trace from a mouse with sham or CLP treatment. (C) The neurobehavioral scores, indicative of mice’s neurological injury, were assessed with a sample size of *n* = 6 mice per group. (D) Percentage of center duration within 5 min in the OFT. *n* = 7 to 10 mice for each group. (E) Representative immunofluorescence images of astrocytes and microglia from mice with sham or CLP treatments, along with assessments of microglia morphology and immunoreactivity. At different magnifications, scale bars were set to 50 and 10 μm. (F) Immunofluorescence of CD11b^+^ microglia (green) in the mouse hippocampal brain slice. Scale bar, 20 μm. (G) Western blot analysis depicting GFAP and CD68 expression levels in the mouse hippocampus (left); the right panel shows quantification of GFAP/GAPDH and CD68/β-actin levels in the hippocampus of sham and CLP groups. *n* = 4 to 5 per group. (H) Representative confocal images revealing microglia–astrocytes interaction after CLP (boxed regions). In the magnified boxed areas (right), both the soma and processes of microglia are shown to directly contact astrocytes. Scale bars, 20 and 10 μm. The middle and right panels display quantitative analysis of Iba1^+^/GFAP^+^ colocalization between the sham and CLP mice groups (*n* = 6 per group). Data are presented as mean ± SD. *P < 0.05; **P < 0.01; ***P < 0.001.

The crosstalk between astrocytes and microglia is crucial in the pathogenesis of central nervous system (CNS) disorders [18,19]. Previous studies reported that microglia play a key role in neuroinflammation during SAE through migration [20]. In order to investigate the interaction between microglia and astrocytes in SAE in vivo, the septic mouse model was generated by cecal ligation and puncture (CLP) [21] (Fig. 1A). Compared to mice of the sham group, mice of the CLP group showed obvious abnormalities of electroencephalogram (EEG) and decreased neurobehavioral scores (Fig. 1B and C), which were monitored to confirm brain injury caused by neuronal lesions in sepsis [1]. Open-field test (OFT) was performed to test the emotional dysfunction of septic mice. Quantification of the movement trajectories indicated that the central duration percentage and the movement distance of CLP mice were significantly decreased, while the edge zone time of CLP mice was increased, compared with those of sham mice (Fig. 1D and Fig. S1). Hippocampal tissue sections from both the sham group and CLP group were analyzed by immunofluorescence staining. Specifically, Iba1 (a marker for microglia), CD11b (an indicator of microglia activation), and glial fibrillary acidic protein (GFAP) (a marker for astrocytes) were employed for this examination. The immunofluorescence area and intensity of astrocytes and microglia were markedly elevated following CLP compared to the sham group (Fig. 1E and F). In the hippocampal tissue of septic mice, Iba1-positive cells exhibited an enlarged cell body and shortened, thickened processes, reminiscent of the characteristic morphology of activated microglia (Fig. 1E). Likewise, septic mice exhibited elevated protein levels of both GFAP and CD68 (Fig. 1G).

Next, we detected the spatial association of astrocytes and microglia in mice by GFAP and Iba1 immunofluorescent signals, respectively. Confocal imaging revealed a significantly increased overlap of astrocytes and microglia in the CLP mice, indicating possible physical interaction between these cells in response to CLP (Fig. 1H). Collectively, these findings revealed that CLP treatment significantly activated astrocytes and microglia in mouse hippocampus, and these 2 types of cells might function synergistically to contribute to the pathogenesis of SAE.

### LPS-treated astrocytes recruited microglia in the microfluidic astrocyte/microglia coculture system

**Fig. 2.**
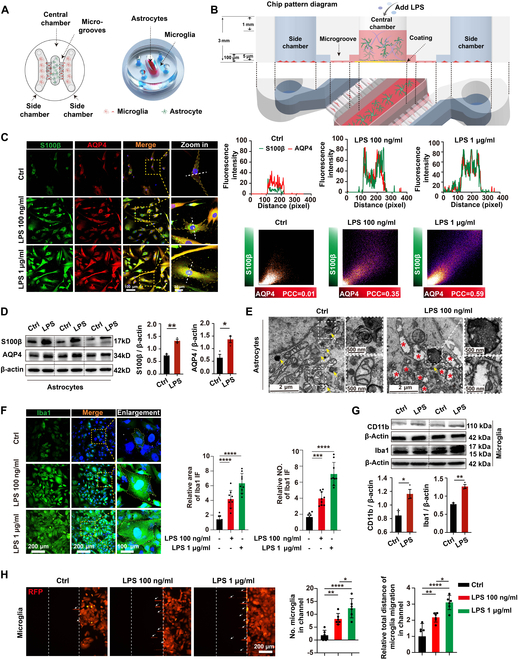
LPS-treated astrocytes recruited microglial cells in the microfluidic astrocyte/microglia coculture system. (A) The microfluidic chamber is labeled with top and overall views, including the central chamber, side chambers, and microgrooves. In the system, the Matrigel-containing central chamber was loaded with human astrocytes. In the side chambers, human microglia labeled with RFP were plated. The central and side chambers are linked with microgrooves (length: 500 μm, width: 10 μm, height: 5 μm). (B) The volume discrepancy between the central and side chambers permits the isolation of distinct chemical microenvironments in the side chamber due to the elevated fluidic resistance of the microgrooves. Conversely, adjusting the volume difference can isolate a specific chemical microenvironment in the central chamber. (C) Human astrocytes were co-immunostained with S100β (green), AQP4 (red), and DAPI (blue), simultaneously; scale bars, 100 and 30 μm; quantitative analysis of S100β and AQP4 intensity and PCC. (D) Representative of Western blot showing the expression of S100β, AQP4, and β-actin in human primary astrocytes. Quantification of S100β/β-actin levels and AQP4/β-actin levels from different groups. *n* = 3 from ≥3 individual chips for each group. (E) Human astrocytes of 2 groups, visualized by TEM. (F) Human microglia were co-immunostained with Iba1 (green) and DAPI (blue), simultaneously; quantitative analysis of relative area and number of Iba1-positive cells. Scale bars, 200 and 100 μm. *n* = 9 regions of interest (ROIs) from ≥3 individual chips for each group. (G) Representative of Western blot showing expression of CD11b, Iba1, and β-actin expression in human microglia. Quantification of CD11b/β-actin levels and Iba1/β-actin levels in the side chambers from different groups. *n* = 3 from ≥3 individual chips for each group. (H) Representative images of microglia-RFP in side chamber and microgrooves. Scale bar, 200 μm. The number of microglia in microgrooves and the relative total distance of microglia migration in microgrooves were analyzed. *n* = 6 ROIs from ≥3 individual chips for each group. Data are mean ± SD. *P < 0.05; **P < 0.01; ***P < 0.001; ****P < 0.0001.

To investigate the interaction between astrocytes and microglia in SAE in vitro, a microfluidic coculture device was designed and fabricated (Fig. S2 and Fig. 2A). The microfluidic device comprises a central compartment and 2 side compartments connected by microgrooves, enabling the culture of astrocytes and microglia separately. To assess the ability of the microfluidic coculture system to replicate a neuroinflammatory response between astrocytes and microglia cells in SAE, we challenged the central chamber with the inflammatory stimulus LPS [22] for 24 h (Fig. 2B). Immunofluorescent staining showed that AQP4 strongly colocalized with S100β in the astrocytes of the central chamber (Fig. 2C). The *R* [Pearson’s correlation coefficients (PCCs)] value and fluorescence intensity of AQP4 and S100β were notably increased by LPS, which proves that human astrocytes were activated in the microfluidic coculture system. Similarly, the S100β and AQP4 protein expression was increased in septic mouse brains (Fig. 2D). Then, we examined the impact of LPS on human astrocytes using transmission electron microscopy (TEM). TEM analysis revealed that damaged mitochondria with broken and reduced cristae were increased, while autophagosomes were decreased, in the LPS group, which were consistent with the pathomorphological changes in SAE (Fig. 2E).

To further evaluate the microglial activation in the microfluidic coculture system, immunofluorescence staining of Iba1 was performed. The relative area and the number of Iba1-positive cells were obviously increased in LPS groups (Fig. 2F). The protein level of CD11b and Iba1 was also elevated when astrocytes were treated with LPS in this system (Fig. 2G). To explore the effect of activated astrocytes on microglia migration in SAE, we labeled microglia with red fluorescent protein (RFP) using a lentivirus vector, and the chemotactic movement of microglia toward astrocytes was observed in the microfluidic coculture system. The microglial number in microgrooves and the relative total distance of microglial migration in microgrooves were increased in the LPS-treated group (Fig. 2H). We also got the same results in different lines of human astrocytes in the microfluidic coculture system (Figs. S3 to S5). Taken together, these findings indicated that activated astrocytes could trigger microglial activation, which facilitated microglial migration toward astrocytes in the microfluidic coculture system.

### Transcriptomic analysis of microglial responses to astrocytic activation in the microfluidic coculture system

**Fig. 3.**
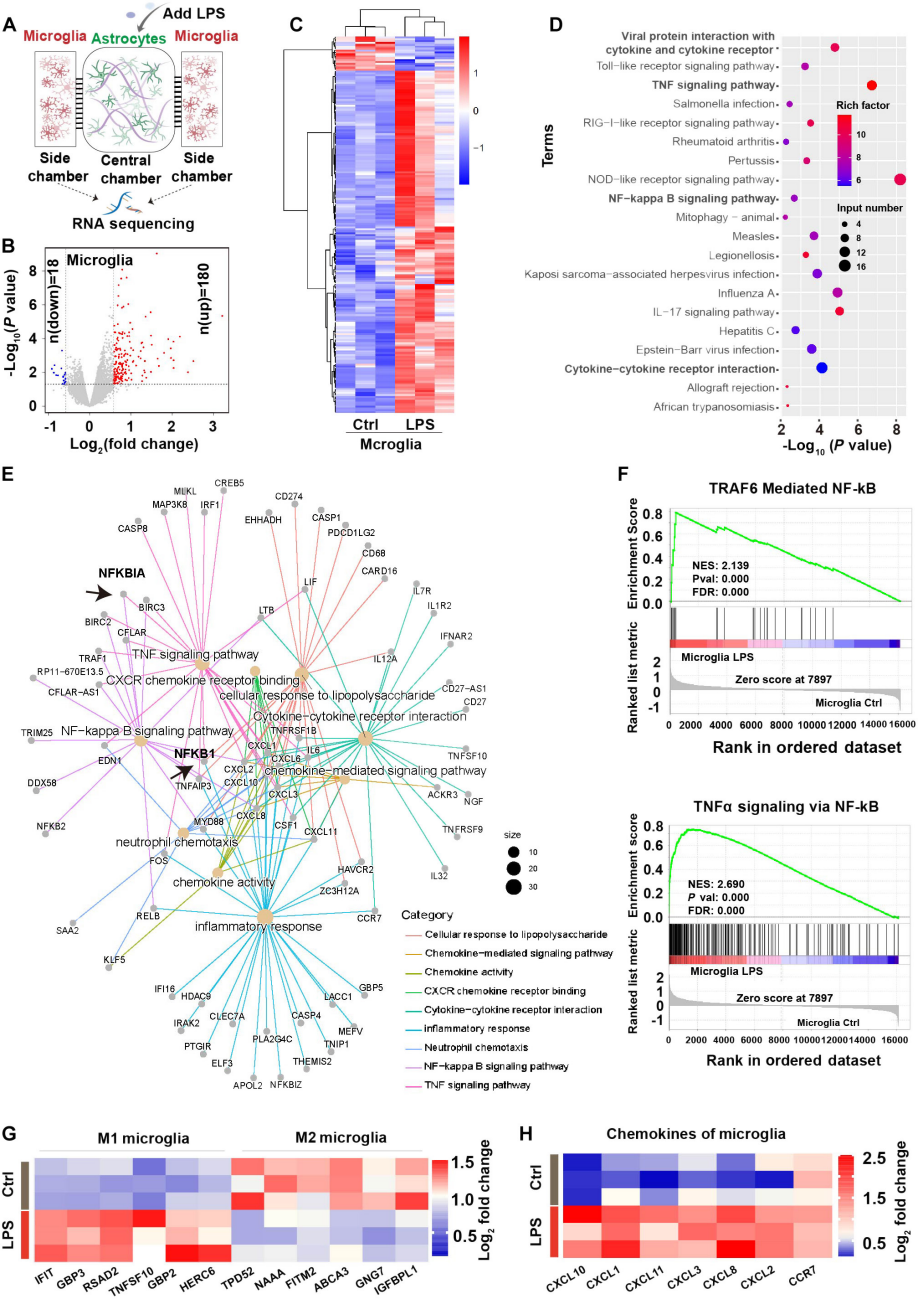
Transcriptional analysis of microglial responses to LPS-treated astrocytes in the microfluidic coculture system. (A) Schematic description of human microfluidic coculture system. (B) Volcano plot of microglia gene expression in a side chamber (*P* < 0.05, |fold change| > 1.5). (C) Heatmap showing transcriptional changes of microglia in the human microfluidic coculture system (*n* = 3). (D) Top 20 pathway analysis, based on the KEGG database, highlights the enrichment of DEGs in side chamber microglia across multiple cellular biological processes. (E) Gene network analysis of KEGG pathways related to inflammatory responses. (F) GSEA analysis revealed that the genes of microglia were mainly enriched in TRAF6-mediated NF-κB and TNF-α signaling via NF-κB-related pathways. (G and H) Heatmaps showing the expression levels of DEGs associated with microglial phenotypes and chemokines in microglia of control and LPS-treated groups (*n* = 3).

To gain a comprehensive overview of the molecular signatures underlying microglial activation induced by activated astrocytes in SAE, we analyzed the transcriptional profiles of microglia in side chamber by RNA sequencing (RNA-seq) (Fig. 3A). Among the differentially expressed genes (DEGs; *P* < 0.05, |fold change| > 1.5), 180 genes were up-regulated and 18 genes were down-regulated in the microglia following LPS treatment for the astrocytes (Fig. 3B). The heatmap showed that LPS-treated astrocytes in the central chamber induced transcriptome modulation in microglia (Fig. 3C) and in astrocytes (Fig. S6).

A more comprehensive analysis of Kyoto Encyclopedia of Genes and Genomes (KEGG) pathways revealed that the biological pathways associated with cytokine–cytokine receptor interactions, the NF-κB signaling pathway, and the tumor necrosis factor (TNF) signaling pathway were modulated in microglia in the LPS-treated group (Fig. 3D). Combined gene network analysis further suggested that NF-κB was involved in pathways including cellular response to LPS, inflammatory response, and NF-κB signaling pathway directly and was involved in pathways including TNF signaling pathway, chemokine-mediated signaling pathway, chemokine activity, CXCR chemokine receptor binding, and cytokine–cytokine receptor interaction indirectly (Fig. 3E). The Gene Set Enrichment Analysis (GSEA) analysis indicated the predominant enrichment of microglial genes within pathways associated with TRAF6-mediated NF-κB signaling and TNF-α signaling via NF-κB (Fig. 3F). The heatmap showed NF-κB signaling pathway-associated transcriptomic modulation in microglia when central astrocytes were treated with LPS (Fig. S7). Microglia, the resident macrophage-like cells in CNS, have 2 opposite phenotypes, M1 (pro-inflammatory phenotype) and M2 (anti-inflammatory phenotype) [23]. Strikingly, LPS treatment in the central chamber led to elevated expression of M1 phenotype-related genes (IFIT, GBP3, RSAD2, TNFSF10, GBP2, and HERC6) and genes critical to chemotactic movement (CXCL10, CXCL1, CXCL11, CXCL3, CXCL8, CXCL2, and CCR7). Meanwhile, LPS treatment in the central chamber led to reduced expression of M2 phenotype-related genes (TPD52, NAAA, FITM2, ABCA3, GNG7, and IGFBPL1) (Fig. 3G and H).

These data indicated that astrocytic activation induced by LPS confers broad and substantial reprogramming of the microglial transcriptome and promotes microglial M1 polarization and chemotactic responses, which might be mediated by the NF-κB signaling pathway.

### Astrocyte-derived IL11 is involved in the microglial response to LPS-induced neuroinflammation in the microfluidic coculture system

**Fig. 4.**
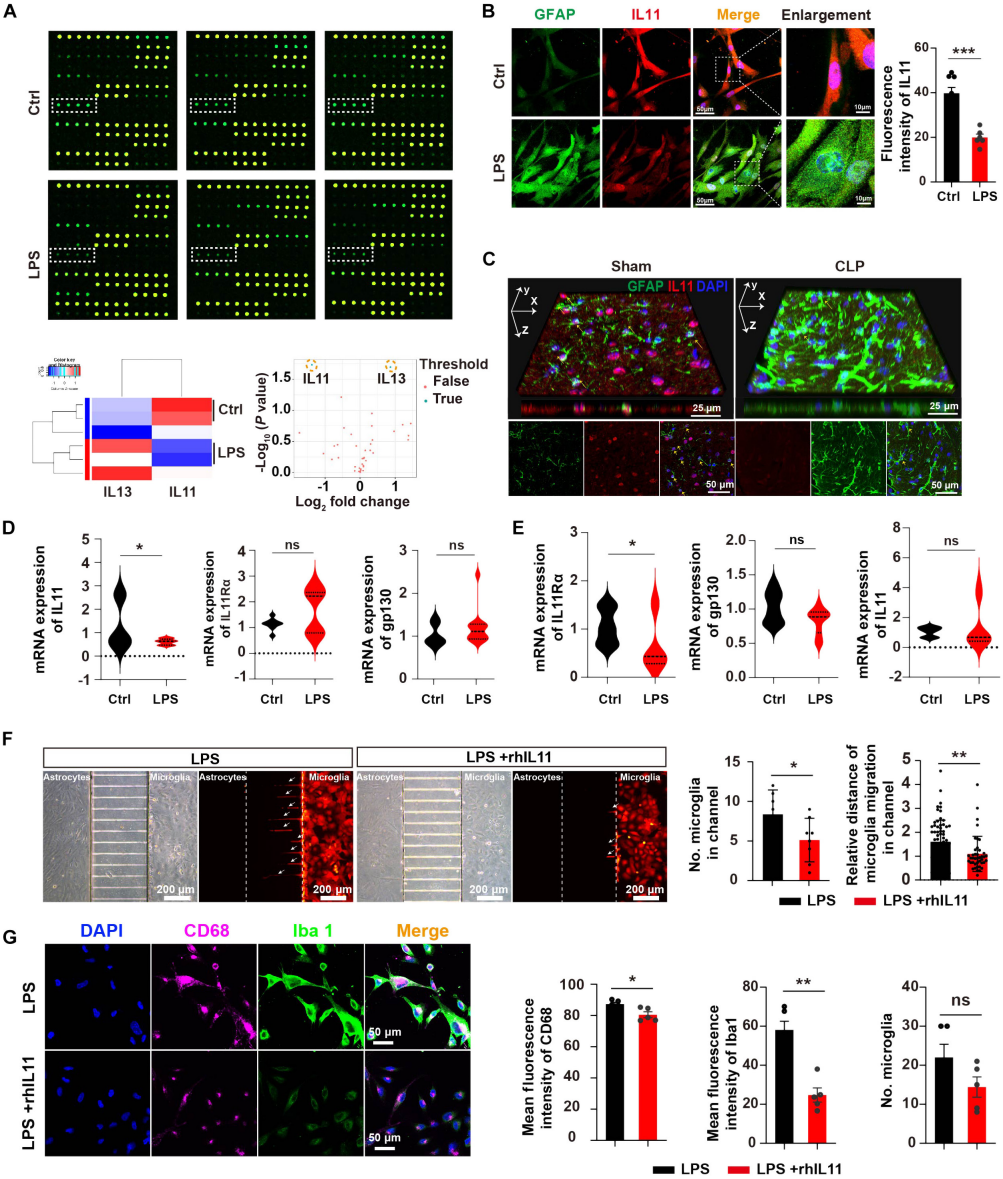
The decreased astrocyte-derived IL11 was implicated in the regulatory process between astrocytes and microglia in the microfluidic coculture system. (A) Cytokine profile. Top: Dots in the white boxes are references (IL11). Bottom: Protein concentration was measured in conditioned media extracted from central chambers with or without LPS. (B) Human astrocytes were co-immunostained with GFAP (green), IL11 (red), and DAPI (blue) in the microfluidic system, simultaneously; scale bars, 50 and 10 μm. The intensity of IL11 was analyzed. *n* = 6 to 8 ROIs from ≥3 individual chips for each group. (C) Distribution of IL11 in astrocytes of mice with or without CLP. Co-immunostaining was conducted with anti-IL11 (red) and anti-GFAP (green) antibodies, along with DAPI for nuclei. Yellow arrows indicate IL11 and GFAP colocalization. A 3-dimensional reconstruction of confocal z-stacks of IL11 and GFAP in mouse brain slices is shown. Scale bars, 25 and 50 μm. (D) mRNA expression levels of IL11, IL11Rα, and gp130 in astrocytes of the central chamber from the control and LPS groups. (E) mRNA expression levels of IL11Rα, gp130, and IL11 in microglia of the side chamber from the control and LPS groups. (F) Representative images of microglia-RFP in side chamber and microgrooves between LPS and LPS + rhIL11 groups. Scale bar, 200 μm. The number of microglia in microgrooves and the relative distance of microglia migration in microgrooves are analyzed. *n* = 8 ROIs from ≥3 individual chips for each group. (G) Human microglia were co-immunostained with Iba1 (green), CD68 (purple), and DAPI (blue), simultaneously; scale bar, 50 μm; quantitative analysis of mean fluorescence intensity of CD68, Iba1, and number of microglia cells between LPS and LPS + rhIL11 groups. *n* = 5 ROIs from ≥3 individual chips for each group. Data are mean ± SD. *P < 0.05; **P < 0.01; ***P < 0.001.

Previous researches reported that astrocytes can manipulate microglial activation and modulate neuroinflammatory responses by secreting cytokines in CNS inflammation [13,24]. Next, we set out to identify secreted cytokines or chemokines from activated astrocytes that regulate microglial responses in SAE. The cell culture supernatant in the central chamber was collected 24 h after LPS treatment and analyzed by RayBiotech chip assay for detection of the cytokines or chemokines. The results showed that the IL13 level was significantly increased, while the IL11 level was significantly down-regulated in the central chamber (Fig. 4A and Fig. S8). Previous study reported that IL13 is an anti-inflammatory cytokine that reduces inflammatory cytokine production in sepsis [25]. Then, we focused on IL11. IL11 belongs to the IL6 cytokine family, and it has been demonstrated that IL11 can influence macrophage function by inhibiting the production of TNF-α, IL1β, and IL12 [26–28]. The IL11 receptor is composed of IL11Rα, responsible for binding IL11, and gp130, which is associated with signal transmission to the nucleus via the Janus kinase (JAK) [29].

Next, we sought to assess IL11 expression in human astrocytes and mouse brains. We observed that IL11 was down-regulated in the astrocytes following LPS treatment (Fig. 4B). In line with our findings in vitro, histology examination revealed that IL11 colocalized with astrocytes in the hippocampus of mice, and astrocyte-derived IL11 decreased significantly 12 h after CLP (Fig. 4C and Fig. S9A and B), and the accumulation of microglia and astrocyte activation occurred 24 h after CLP (Fig. S9C and D). These results, together with our previous data, suggested that down-regulation of IL11 expression in astrocytes is a major trigger for microglial activation. Besides, quantitative polymerase chain reaction (qPCR) analysis showed that LPS treatment reduced IL11 mRNA levels in astrocytes, with no significant change in IL11Rα and gp130 mRNA levels between the control and LPS groups (Fig. 4D). In microglia, LPS treatment reduced IL11Rα mRNA levels, but IL11 and gp130 mRNA levels remained unchanged between the 2 groups (Fig. 4E). We then quantified recruitment of RFP-labeled human microglia to the astrocytes induced by LPS in the central chamber using a confocal microscope (Fig. 4F). The result showed that microglia recruitment was augmented in cultures containing LPS-stimulated astrocytes, while rhIL11 (recombinant human IL11) addition robustly decreased microglia migration from side chambers to the central chamber (Fig. 4F) and alleviated the microglial activation (Fig. 4G). Collectively, these results indicated that activated astrocytes secreted less IL11, which in turn induced microglial activation and migration to the region of neural injury in SAE.

### Decreased astrocyte-derived IL11 induced microglial activation and migration via NF-κB signaling pathway underlying SAE pathogenesis

**Fig. 5.**
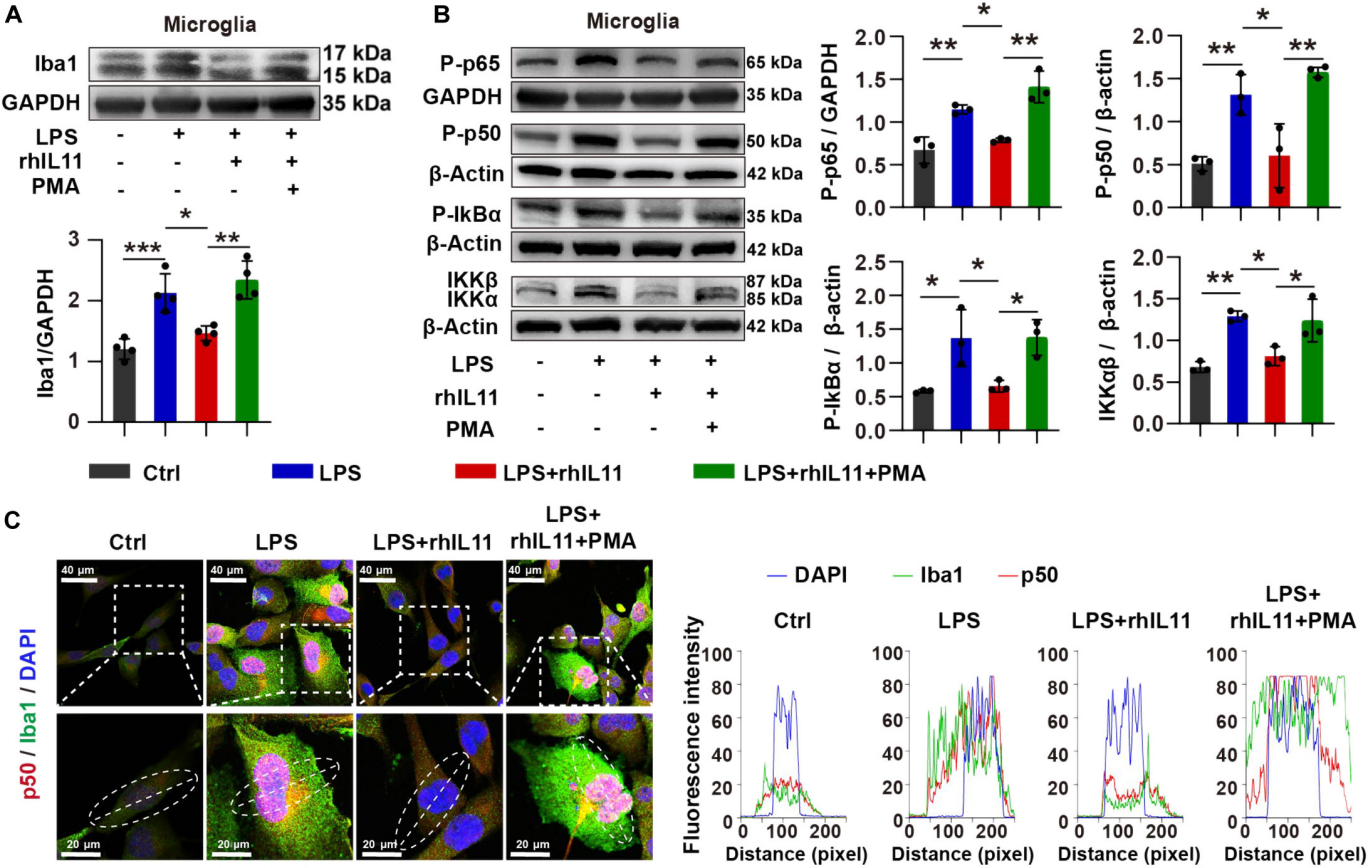
IL11 treatment alleviated microglia activation by inhibiting the NF-κB signaling pathway. (A) Western blot showing the protein levels of Iba1 in 4 groups. *n* = 4 from ≥3 individual chips for each group. (B) Western blot showing the protein levels of NF-κB signaling pathway-related protein. *n* = 3 from ≥3 individual chips for each group. (C) Confocal micrographs of microglia in the side chamber, double-stained with anti-p50 (red) and anti-Iba1 (green) antibodies. Enlarged views of the white-boxed areas are shown at the bottom left. Line-scan analysis of p50 (red) and Iba1 (green) is provided along the dotted white lines. Scale bar, 20 μm. Data are presented as mean ± SD. *P < 0.05; **P < 0.01.

Previous studies report that the astrocyte–microglia crosstalk mediates neuroinflammation, which contributes to neuronal injuries in some CNS diseases [30,31]. To detect the specific molecular mechanisms of the astrocyte–microglia crosstalk in regulating neurons in SAE, a microfluidic neuron/astrocyte/microglia tri-culture system that recapitulates neuropathogenesis associated with SAE was established to detect the effect of astrocyte-derived IL11 on microglia activation and neuronal injury (Fig. S10). Human astrocytes and neurons, both with and without exposure to LPS, are seeded within the central chamber, whereas human microglia labeled with RFP are seeded in the side chambers. The Western blotting (WB) results showed that LPS-induced increase of Iba1 was reversed by rhIL11 treatment. However, phorbol 12-myristate 13-acetate (PMA), which is a commonly used NF-κB activator, mitigated the effect of rhIL11 (Fig. 5A). CD68 expression was significantly elevated, and C206 expression was significantly decreased when the central chamber was induced with LPS, while it was reversed by rhIL11 treatment (Fig. S11). These results suggest that rhIL11 reduced M1 polarization of microglia and switched them from M1 to M2 polarization.

To further assess whether the microglial activation and migration toward astrocytes was caused by the activation of the NF-κB signaling pathway, we performed WB and double immunofluorescence staining of Iba1 and NF-κB in the side chamber of the microfluidic tri-culture system. The WB results showed that the protein level of P-p65, P-p50, P-IκBα, and IKKα/β were elevated when the central chamber was induced with LPS. However, rhIL11 alleviated the expression increase of P-p65, P-p50, P-IκBα, and IKKα/β induced by LPS, and PMA mitigated the effect of rhIL11 (Fig. 5B). Astrocytes exposed to LPS led to a heightened degree of colocalization between p50 and 4′,6-diamidino-2-phenylindole (DAPI) in microglia while simultaneously increasing the intensity of Iba1 in microglia. However, the increased level of colocalization of p50 with nuclear DAPI induced by LPS was alleviated when the central chamber cells were treated with rhIL11. Furthermore, PMA antagonized the effect of rhIL11 on this system (Fig. 5C).

These above findings indicated that decreased IL11 derived from astrocytes could induce microglial activation via the NF-κB signaling pathway in SAE.

### IL11 administration ameliorated neuronal injury in SAE models

**Fig. 6.**
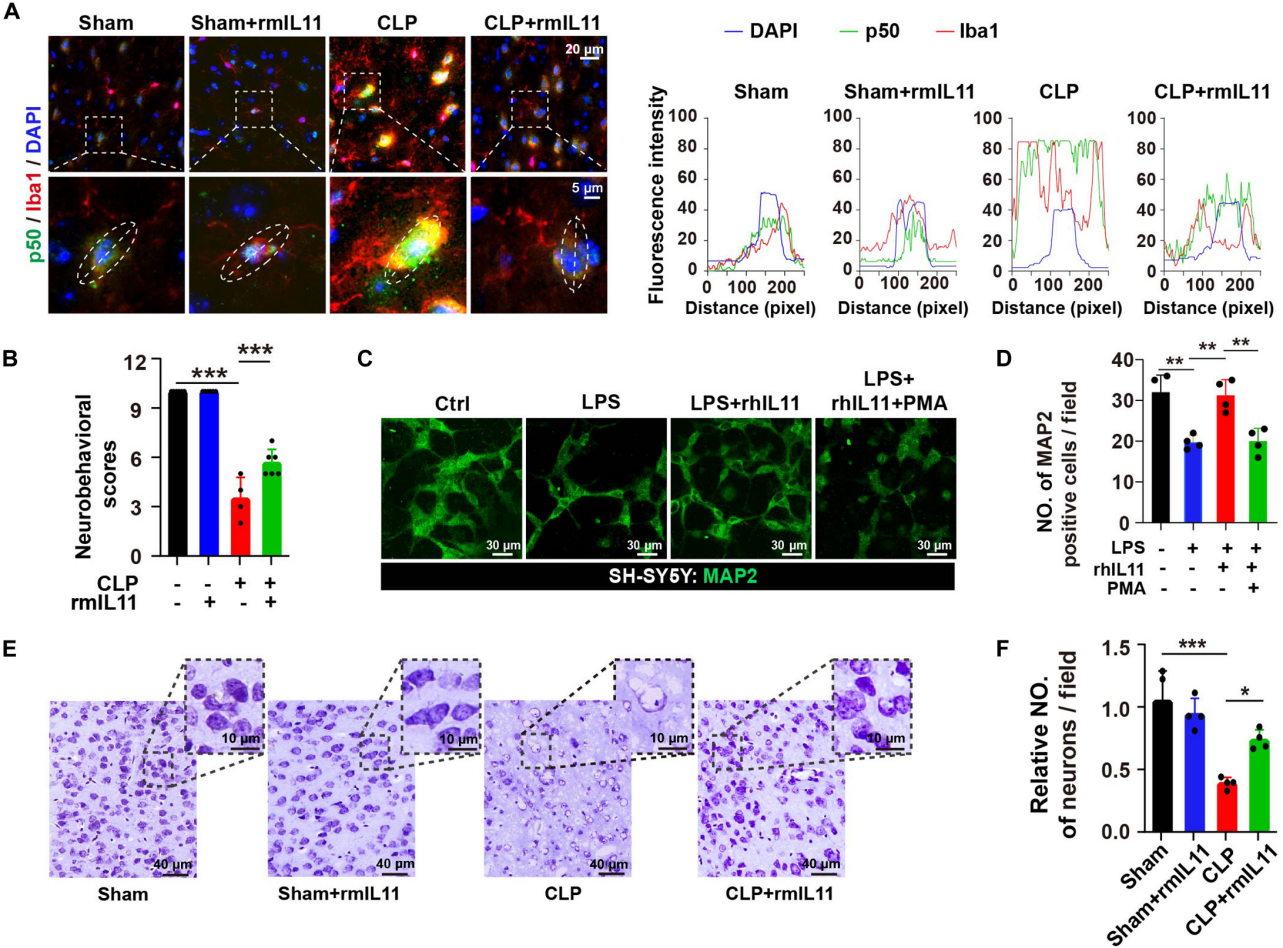
IL11 administration ameliorated neuronal injury in SAE models. (A) Confocal micrographs of microglia in mouse brain, double-stained with anti-p50 (green) and anti-Iba1 (red) antibodies. Enlarged views of the areas marked by white boxes are displayed at the bottom left. Line-scan analysis of p50 (red) and Iba1 (green) along the dotted white lines. (B) Neurobehavioral scores reflecting the neurological status of mice (*n* = 4 to 6 mice per group). (C) Human neurons in the central chamber were immunostained for MAP2 (green). The central chamber was stimulated with rhIL11 and LPS challenge, and the side chamber was stimulated with PMA. Scale bar, 30 μm. (D) Quantitative analysis of the number of MAP2-positive cells in different groups. *n* = 4 ROIs from ≥3 individual chips for each group. (E) Representative images of Nissl-stained hippocampal sections from various experimental groups. Scale bar, 10 and 40 μm. (F) Quantitative analysis of a relative number of neurons per field in different groups. *n* = 4 ROIs from ≥3 mice for each group. *P < 0.05; **P < 0.01; ***P < 0.001.

To evaluate the potential therapeutic value of IL11 in treating SAE, sham and CLP mice received intraperitoneal injection of phosphate-buffered saline (PBS) or rmIL11 (recombinant mouse IL11). Double immunofluorescence staining of p50 and Iba1 in mouse brain slices was performed and showed similar results to that of the microfluidic tri-culture system (Fig. 6A). The neurobehavioral results of the mice with CLP and rmIL11 had higher scores than those of mice with CLP only (Fig. 6B). To dissect the roles of microglia in the neuronal injury of SAE, we also performed an ablation study of microglia on the neuron/astrocyte/microglia tri-culture system. The results showed that LPS treatment induced obvious cell apoptosis [terminal deoxynucleotidyl transferase–mediated deoxyuridine triphosphate nick end labeling (TUNEL)-positive cells] in the neuron/astrocyte culture compartment [SH-SY5Y cells were labeled by green fluorescent protein (GFP)], while ablation of microglia (without microglia) partially ameliorated the LPS-induced apoptosis, which indicated that activated microglia played a detrimental effect on the neuronal cells in the pathogenesis of SAE (Fig. S12). To further investigate the mechanism that IL11 modulates microglial function to protect neurons in SAE, rhIL11 and PMA were added to the central chamber of the microfluidic tri-culture system. Confocal imaging showed that rhIL11 rescued the neuronal injury induced by LPS; however, PMA mitigated the rescue effect of rhIL11 (Fig. 6C and D). Cresyl violet staining that enables the assessment of neuronal cell death was performed in the CLP model. We observed disorganized cellular structures with contracted cytoplasm and perineuronal vacuoles containing darkened nuclei in the CLP group, which were markedly ameliorated following rmIL11 treatment (Fig. 6E and F).

**Fig. 7.**
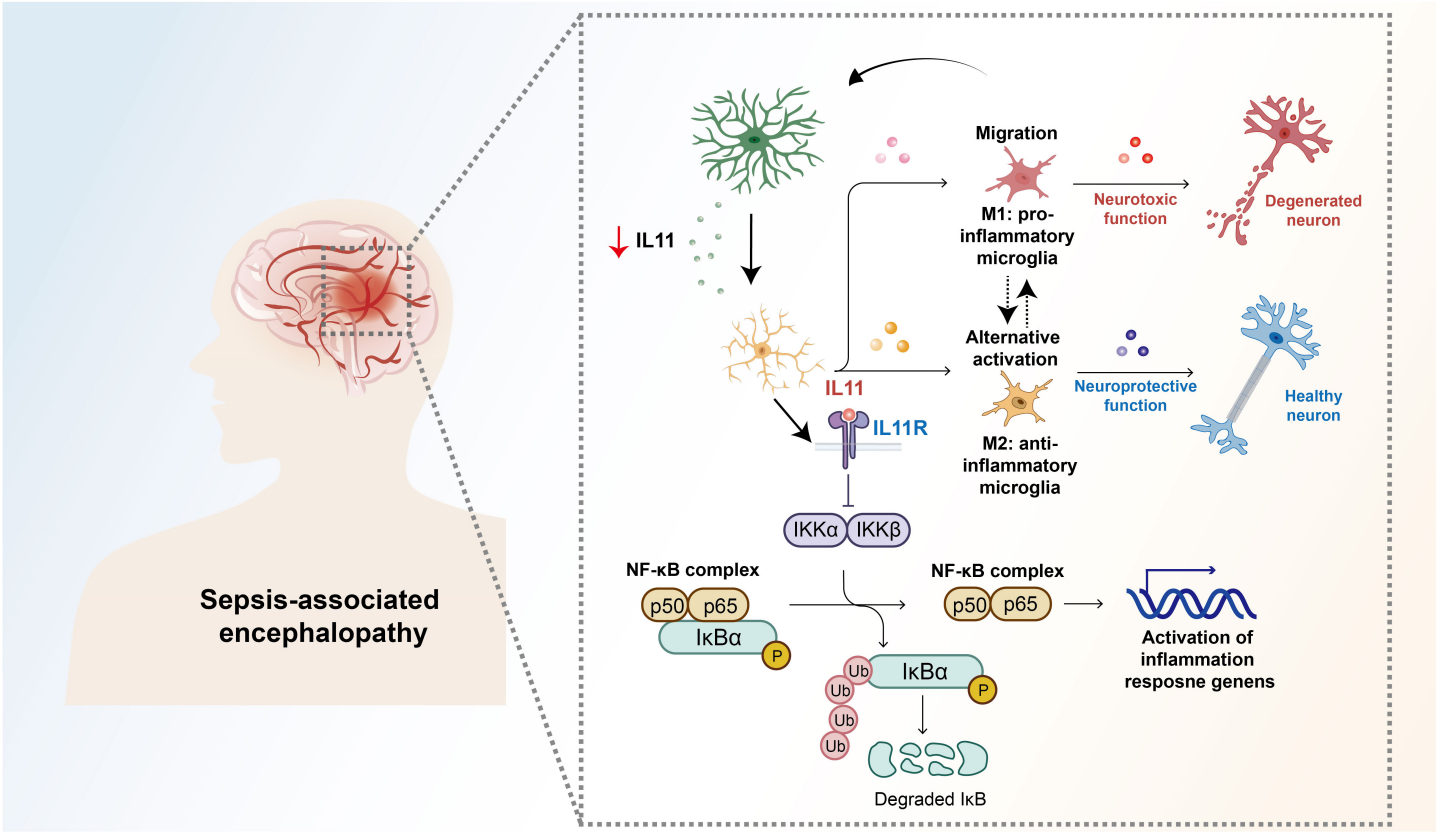
Proposed mechanism of astrocyte-derived IL11 mediating astrocyte–microglia crosstalk via the NF-κB signaling pathway in SAE. In SAE, decreased astrocyte-derived IL11 results in microglial activation with enhanced M1 polarization and increased migration, which is medicated by the NF-κB signaling pathway. Astrocyte-derived IL11 decreases, which causes the activation of the IKK complex and degradation of IκBα. This results in the translocation of the NF-κB complex (p50, p65) into the nucleus, triggering gene expression changes associated with inflammation, including microglia polarization and migration-related genes, while IL11 administration can inhibit the microglial activation caused by the decrease of astrocyte-derived IL11 and ameliorate the neuronal injury in SAE.

These results suggest that IL11 addition can reverse microglial activation by inhibiting the NF-κB signaling pathway to alleviate neuronal injury in SAE. The suggested mechanisms by which astrocyte-derived IL11 mitigates neuronal damage have been outlined in Fig. 7.

## Discussion

Excessive glial activation that occurred in SAE has raised great concerns [32,33], and higher levels of glial activation were associated with more severe neuronal damage in SAE [34,35]. Our previous work reported that astrocyte activation and inflammatory cytokine response strongly contributed to the progression of neuronal injury during SAE [1]. This study expands our understanding of the potential role of astrocytes in SAE [1]. However, the roles of microglia and the potential synergistic effect between microglia and astrocytes in SAE remain unclear. Here, we presented a new human astrocyte/microglia coculture system on a microfluidic device to model neuroinflammation in SAE, which enables us to explore the crosstalk between astrocytes and microglia during the disease pathogenesis.

With regard to the astrocyte–microglia crosstalk, previous studies have reported that it is involved in the pathogenesis of many neurological diseases, including Parkinson’s disease (PD) [36], multiple sclerosis [37], epilepticus [18], and so on [38]. However, the glial cell communication in the pathophysiology of SAE remains poorly defined. Our study provided key insights into the molecular basis of the communication between astrocytes and microglial cells. The in vivo data showed that both astrocytes and microglia were activated and resided closely in the SAE mouse model. Consistently, we also detected obvious microglial activation and recruitment by activated astrocytes following LPS treatment in the human microfluidic coculture system. Microglia, the resident immune cells of the CNS, are essential for preserving brain homeostasis. However, microglial excessive activation can damage other parenchyma cells and healthy neurons in some pathological conditions [39,40]. Strikingly, our RNA-seq results showed increased expression of M1 phenotype-related genes and decreased expression of M2 phenotype-related genes in microglia when astrocytes were induced by LPS. This indicates that microglia activation follows astrocyte activation in SAE. Microglia, the primary immune defense in the CNS, are recruited to injury or infection sites to clear cell debris and participate in regeneration. GSEA and KEGG pathway analyses revealed that microglia-related genes were mainly enriched in NF-κB signaling pathways. Increasing evidence assigns that glial cell interactions disrupt astrocyte–microglia–neuron communication, which, in turn, puts microglia in a deleterious reactive state that generally increases neuronal damage in neuroinflammation [30]. These findings align with prior researches that microglial recruitment was augmented and genes critical to cell motility were significantly increased following the astrocytes induced with LPS [41–43].

Cytokines, key components of the immune system, mediate most astrocyte–microglia interactions in CNS inflammation [19,44–46]. Recent studies showed that astrocyte-secreted cytokines, such as IL3 and IL33, control microglia recruitment and activation, leading to astrocyte pathogenic activation and neuronal injury in AD and EAE (experimental autoimmune encephalomyelitis) subsequentially [13,30]. In our study, we discovered that IL11 is a vital messenger molecule that mediated the astrocyte–microglia crosstalk underlying SAE pathogenesis. Generally, IL11 is recognized for its anti-inflammatory properties, by inhibiting the release of nitric oxide (NO), TNF-α, IL1β, IL12, IL10, transforming growth factor β (TGFβ), and IL6 mediated by LPS, and directly antagonizing macrophage/monocyte-induced TNF-α signaling [47,48]. A previous research identified a reduction in the IL11 level in the context of cerebral ischemia–reperfusion injury [49], while administration of IL11 notably mitigated the activation of astrocytes and microglia induced by ischemia and also reduced the production of pro-inflammatory cytokines [49]. In this study, we found that, in response to inflammatory stimuli, reactive astrocytes secreted less IL11, which, in turn, activated microglia and fostered microglial migration to the region of neural injury. Reflecting in vivo data, these results obtained with human glial cells point to a critical role for IL11 in microglial recruitment toward human astrocytes in SAE. Especially, we found that IL11 addition notably rescued LPS-induced neuronal injuries on microfluidic system and brain injury in the SAE mouse model.

However, the exact role of astrocyte-derived IL11 in microglia during SAE remains unknown. Studies show that IL11R is expressed in microglia, and their activity is regulated by extracellular signals, including cytokines from the IL11/gp130 family [50,51]. The assembly of ligand–receptor–gp130 complexes triggers a signal transduction pathway that inhibits NF-κB signaling [52]. Accumulating studies have revealed that NF-κB signaling is a vital modulator in the process of microglia activation and polarization, which contributes to neuronal injuries via excessive neuroinflammation [42,43]. We therefore hypothesized that IL11 as an astrocyte-derived factor might regulate the NF-κB signaling pathway to mediate microglia activation and migration in the SAE. First, the WB results showed that LPS-induced Iba1 increase was alleviated by rhIL11. However, PMA mitigated the effect of rhIL11. Further, in the signaling pathway leading to NF-κB activation, its inhibitor protein, IKBα, undergoes phosphorylation by IKKα/β, followed by ubiquitination or proteolytic degradation. Consequently, our findings indicate that rhIL11 suppresses the phosphorylation of NF-κB1 (p50), RelA (p65), and NF-κBIA (IKBα), as well as the nuclear translocation of p50 subunits. The NF-κB activator PMA partially reversed these effects of IL11. These results indicated that astrocyte-derived IL11 suppressed microglial activation through the NF-κB signaling pathway in SAE.

SAE is a severe neurological disease caused by bacterial infection for which there is no effective cure until now; therefore, it is clinically vital to develop therapeutics for this disease. In this study, we discovered that IL11 is a vital messenger factor that mediated astrocyte–microglia underlying the pathogenesis of SAE. In response to pathological stimuli underlying SAE, reactive astrocytes secreted less IL11, which, in turn, led to activation and increased mobility in microglia via the NF-κB signaling pathway. Besides, in vivo CLP mouse model also showed similar results. In particular, we found that IL11 addition could effectively inhibit the overactivation of microglia and rescue the neuronal injury in both in vitro and in vivo models. Collectively, our study indicated that the decreased IL11 level could be used as a risk indicator clinically, and IL11 addition might be a new countermeasure therapeutic for this devastating disease.

At present, microfluidic chips have emerged as valuable tools for cell biology due to their capacity for precise control, monitoring, and manipulation of cellular microenvironments [13,41,53–55]. In this study, we established a microfluidic astrocyte/microglia coculture system to provide a new method to visualize, isolate, and biochemically analyze glial cell interaction [13,56]. Astrocytes and microglia can be cultured in separate chambers in the microfluidic chamber. Then, we can analyze the interaction between astrocytes and microglia underlying neuroinflammation by introducing LPS into the central chamber, which was already seeded with astrocytes and neurons. This innovative microfluidic chip-based platform allowed us to faithfully replicate the complex dynamics of SAE in an in vitro setting, which can also be utilized for development of new therapeutics.

In summary, this work made the first attempt to establish a human microfluidic astrocyte/microglia coculture system to analyze the intercellular communications in SAE. Based on the platform, we found that activated astrocytes recruit microglia in SAE. Astrocyte-derived IL11 modulates the inflammatory state of microglia via the NF-κB signaling pathway in this disease. Our study defines an unknown astrocyte-to-microglia crosstalk mediator IL11, representing a promising therapeutic target for mitigating the detrimental effects on neurons in SAE.

## Materials and Methods

### Animal studies

Male C57BL/6 mice (8 to 10 weeks old, 20 to 25 g) were obtained from the Specific Pathogen-Free (SPF) Model Animal Center of Dalian Medical University. Animal experiments were conducted in accordance with the ARRIVE guidelines to ensure rigorous reporting and ethical standards [57,58]. The mice were given at least 1 week to acclimate before the experiments began. Afterward, they were randomly assigned to groups and provided with appropriate housing.

### Establishment of CLP model

SAE was induced using the CLP method [21], with slight modifications. Mice were used in the CLP model with an overall mortality rate of ~70%. The neurobehavioral scores, EEG, and Nissl staining are conducted as previously reported [1]. Ten minutes before sham or CLP surgery, the mice received intraperitoneal injections of either 20 μg/kg rmIL11 (PeproTech, #220-11) [49] or an equivalent volume of 0.9% physiological saline, with sham and CLP mice treated accordingly. The mice were euthanized 24 h after either the CLP or sham surgical procedures, and then the mouse brain was collected after euthanization.

### Open-field test

The OFT was conducted in a quiet room with a stable temperature. The arena measured 50 × 50 cm, with peripheral walls 50 cm high. The central area was defined as the innermost 9 squares of the 25-square grid. Each mouse was allowed to explore the arena for 5 min, and the percentage of time spent in the central area was calculated as (time spent in the central area/300 s) × 100%.

### Immunofluorescence

Immunofluorescent staining of mouse brain slices was conducted as previously [1]. Immunofluorescence imaging of microfluidic chips was conducted as follows: Cells in the side and central chambers were fixed with 4% paraformaldehyde. Subsequently, the cells were treated to achieve permeabilization and were then blocked using a buffer consisting of 0.2% Triton X-100 in PBS (PBST) along with 5% normal goat serum for 30 min at room temperature. The primary antibodies were diluted in 2% PBST buffer supplemented with 5% normal goat serum. Cells were subjected to staining with primary antibodies overnight at 4 °C, followed by the application of corresponding secondary antibodies at room temperature for 1 h in a light-protected environment. The cells were counterstained with DAPI. Finally, anti-fluorescence quenching was added to this system for images acquired using an Olympus FV3000.

### Western blot

Protein samples from both mice and cells were resolved on sodium dodecyl sulfate–polyacrylamide gel electrophoresis (SDS-PAGE) gels and transferred onto polyvinylidene fluoride (PVDF) membranes. Membranes were incubated with primary antibodies overnight at 4 °C, followed by secondary antibody incubation. Band signals were detected using electrochemiluminescence (ECL) reagent from Millipore, and gel analysis was performed using Bio-Rad software (Hercules, CA, USA).

### Cell culture

HA cells (human primary astrocytes, catalog no. 1800) were obtained from ScienCell and cultured in AM medium (catalog no. 1801). HPA cells (human primary astrocytes, catalog no. Pri-iCell-007) were purchased from iCell and cultured similarly in AM medium. Human astrocyte U251 cells (catalog no. U251) were acquired from Procell and cultured in Dulbecco’s modified Eagle’s medium (DMEM) (Gibco, catalog no. C11995500BT) with 10% fetal bovine serum (FBS) (Gibco, catalog no. 10099-141) and 1% penicillin–streptomycin (Gibco, catalog no. 15140-122). HMC3 microglial cells (catalog no. CL-0620) were sourced from Procell and grown in MEM (Procell, catalog no. PM150410) with 10% FBS and 1% penicillin–streptomycin. SH-SY5Y neuroblastoma cells, a gift from B. Zhao (Dalian Institute of Chemical Physics), were cultured in DMEM/F12 (Gibco, catalog no. C11330500BT) with 10% FBS and 1% penicillin–streptomycin. To induce neuronal differentiation, SH-SY5Y cells were treated with 10 μM all-trans retinoic acid (RA) (Sigma, catalog no. 390900) for 5 d. GFP-expressing SH-SY5Y and RFP-expressing HMC3 stable cell lines were generated by lentiviral transfection and selected using 5 μg/ml puromycin (Beyotime, catalog no. ST551).

### Microfluidic device fabrication

Microfluidic devices were fabricated by patterning SU-8 10 and SU-8 100 (MicroChem, USA) on a 4-inch silicon wafer using standard lithography, forming molds with cell migration channels (500 μm length, 10 μm width, 5 μm height) and side chambers. A 10:1 mixture of SYLGARD 184 base and curing agent was poured onto the SU-8 mold and cured for 1 h at 25 °C under vacuum and then for 3 h at 80 °C. After detaching the polydimethylsiloxane (PDMS) replica, 3.5/1.6-mm holes were punched for cell chambers. PDMS was bonded to glass slides via oxygen plasma treatment (150 mW, 30 s), followed by 1-h curing at 45 °C. Chips were sterilized with ultraviolet light, and channels were precoated with 0.2% collagen type I at 37 °C overnight. In the coculture device, HA cells (1 × 10^6^ cells/ml) were seeded into the central channel. For tri-culture, HA and SH-SY5Y cells (1:1 ratio, 1 × 10^6^ cells/ml) were seeded. After 24 h of stabilization, HMC3 cells (5 × 10^6^ cells/ml) were introduced into the side chambers.

### Drugs

Cells in the central chamber were pretreated with rhIL11 (PEPROTECH, # 200-11-10ug) or LPS (Millipore, #437627) for 24 h. The microglia in the side chamber were pretreated with PMA (MCE, HY-18739).

### Transmission electron microscopy

A microfluidic chip was fixed on the top of an aclar film (Electron Microscopy Sciences, #50425). The microfluidic chips were preserved overnight at 4 °C in electron microscopy fixative (Servicebio, #G1102). Then, the electron microscopy was done according to protocols as previously [1]. The JEM-1400PLUS electron microscope was used to detect the images.

### Cytokine array

Culture supernatant in the central chamber was collected. Cytokines in the culture supernatants were assessed using a quantibody human inflammation array 3 in strict accordance with the manufacturer’s guidelines (RayBiotech, #QAH-INF-3).

### RNA-seq and data analysis

Cells in the chip’s central and side chambers were lysed with 20 μl of TRIzol reagent (Invitrogen), and RNA was extracted and treated with deoxyribonuclease I (DNase I). RNA quality was assessed using a NanoDrop One spectrophotometer for *A*_260_/*A*_280_ ratios and 1.5% agarose gel electrophoresis. RNA was quantified using Qubit 3.0, and 2 μg of total RNA was utilized for stranded RNA-seq library preparation with the KC-Digital Stranded mRNA Library Prep Kit (Wuhan Seqhealth), incorporating unique molecular identifiers to minimize PCR and sequencing bias. Libraries were enriched for 200- to 500-base pair fragments and sequenced on a DNBSEQ-T7 (MGI Tech, China). Sequencing data were filtered with Trimmomatic (v0.36) to remove adaptor sequences. Clean reads, clustered by unique molecular identifiers, underwent pairwise alignment to form subclusters based on >95% sequence identity. Consensus sequences were generated to reduce errors and aligned to the Homo sapiens reference genome (Ensembl release-87) using STAR (v2.5.3a). Gene expression was quantified using featureCounts (Subread-1.5.1) and expressed as reads per kilobase of transcript per million mapped reads (RPKM). Differential expression analysis was conducted with edgeR (v3.12.1) with *P* < 0.05 and fold change > 2. Gene Ontology (GO) and KEGG enrichment analyses were performed with KOBAS (v2.1.1) using *P* < 0.05 as the significance threshold.

### Quantitative real-time PCR

Quantitative real-time PCR was carried out following established protocols as previously described [59]. The primers for IL11, IL11Rα, gp130, and glyceraldehyde-3-phosphate dehydrogenase (GAPDH) (Sangon Biotech, Shanghai, China) are detailed in Table S3.

### Statistical analysis

Graphs were generated using Prism 8.4.2 (GraphPad Software). Normality tests were conducted, and statistical significance was determined as follows: unpaired Student’s *t* tests and Mann–Whitney *U* tests for 2 groups and one-way analysis of variance (ANOVA) analysis with Tukey’s post hoc test for more than 2 groups. A significance level of *P* < 0.05 was applied for all analyses.

## Ethical Approval

All animal procedures adhered to the guidelines of the NIH Institutional Animal Care and Use Committee, USA (NIH Publication No. 86-23, revised 1987) and were approved by the Institutional Ethics Committee of Dalian Medical University (approval no. AEE23052).

## Data Availability

The authors verify the inclusion of all supportive data. Raw sequence data from this study are archived in the Sequence Read Archive via the accession number SUB14016701. Requests for any extra materials linked to this article will be accommodated upon reasonable inquiry directed to the corresponding authors.
