# Supplementary material for: Astrocyte-Derived Interleukin 11 Modulates Astrocyte–Microglia Crosstalk via Nuclear Factor-κB Signaling Pathway in Sepsis-Associated Encephalopathy
Source: Research (Wash D C). 2025 Jan 30;8:0598. doi: 10.34133/research.0598 (PMC11780073; doi:10.34133/research.0598)
Supplement: Supplementary 1 — Figs. S1 to S12 Tables S1 to S3 [file research.0598.f1.docx]

**Supplementary Materials**

**Astrocyte-derived IL11 modulates astrocytes-microglia crosstalk via NF-κB signaling pathway in sepsis-associated encephalopathy**

**Dandan Zhu^1,5,†^, Peng Wang^2,3,†^, Xiyue Chen^1^, Kaituo Wang^1^, Yunsong Wu^1^, Min Zhang^1^, Jianhua Qin^1,2,3,4,^***

^1^Division of Biotechnology, Dalian Institute of Chemical Physics, Chinese Academy of Sciences, Dalian 116023, China

^2^University of Science and Technology of China, Hefei 230026, China

^3^Suzhou Institute for Advanced Research, University of Science and Technology of China, Suzhou 215123, China

^4^Beijing Institute for Stem Cell and Regenerative Medicine, Chinese Academy of Sciences, Beijing 100000, China

^5^Department of Critical Care Medicine, the Second Hospital of Dalian Medical University, Dalian, 116023, China

^†^These authors contributed equally

* Correspondence: Jianhua Qin, Division of Biotechnology, Dalian Institute of Chemical Physics, Chinese Academy of Sciences, 457 Zhongshan Road, Dalian 116023, China. E-mail: jhqin@dicp.ac.cn. Fax: 86-411-84379059.


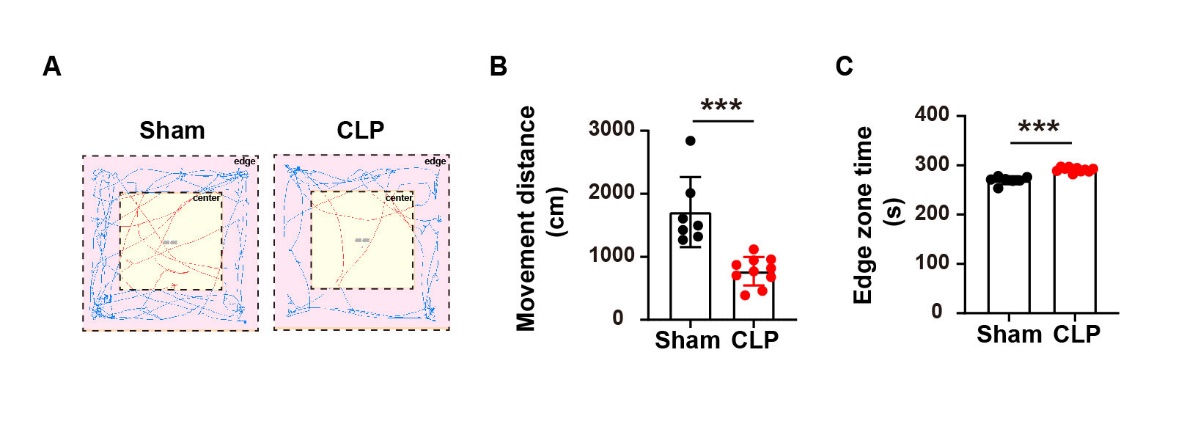
**Supplementary figures**

**Supplementary Fig. 1 | Open field test for sham and CLP mice. A.** Representative movement trajectories for sham and CLP mice (n=7-10). **B**. Quantification of the movement distance for sham and CLP mice. **C.** Quantification of the edge zone time for sham and CLP mice. Data are presented as mean ± SD. ***p < 0.001.


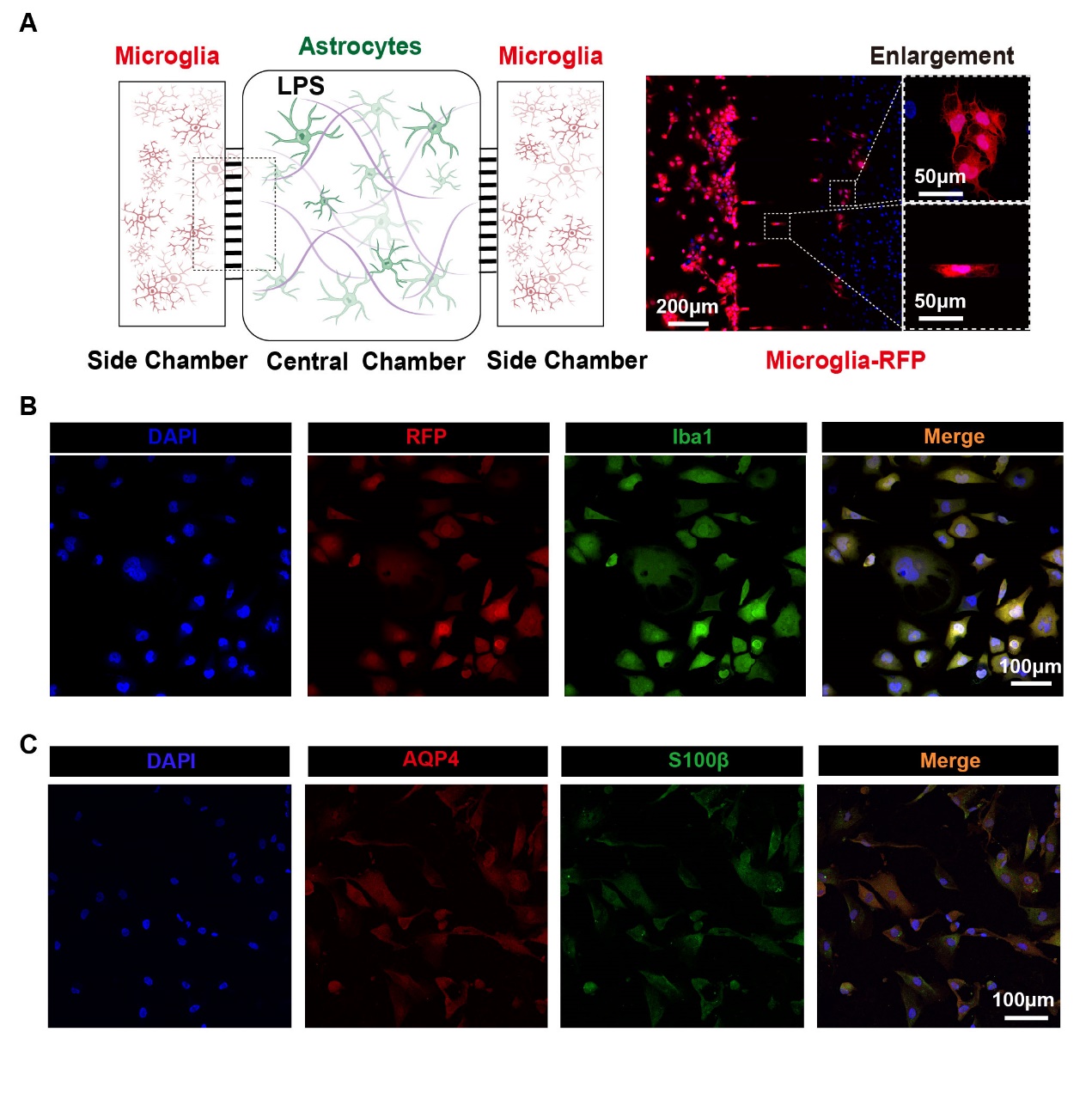
**Supplementary Fig. 2 | Establishment of human microfluidic co-culture system. A**. Scheme of human SAE microfluidic co-culture system, with human astrocytes plated in the central chamber and human microglial cells (labeled with RFP) plated in side chambers. **B**. Immunofluorescent images showing human microglia immunostained with Iba1 (green), and labeled with RFP in the microfluidic co-culture system. Scale bar, 100μm. **C**. Immunofluorescent images showing human astrocytes in the microfluidic co-culture system immunostained with S100β (green) and AQP4 (red). Scale bar, 100μm.


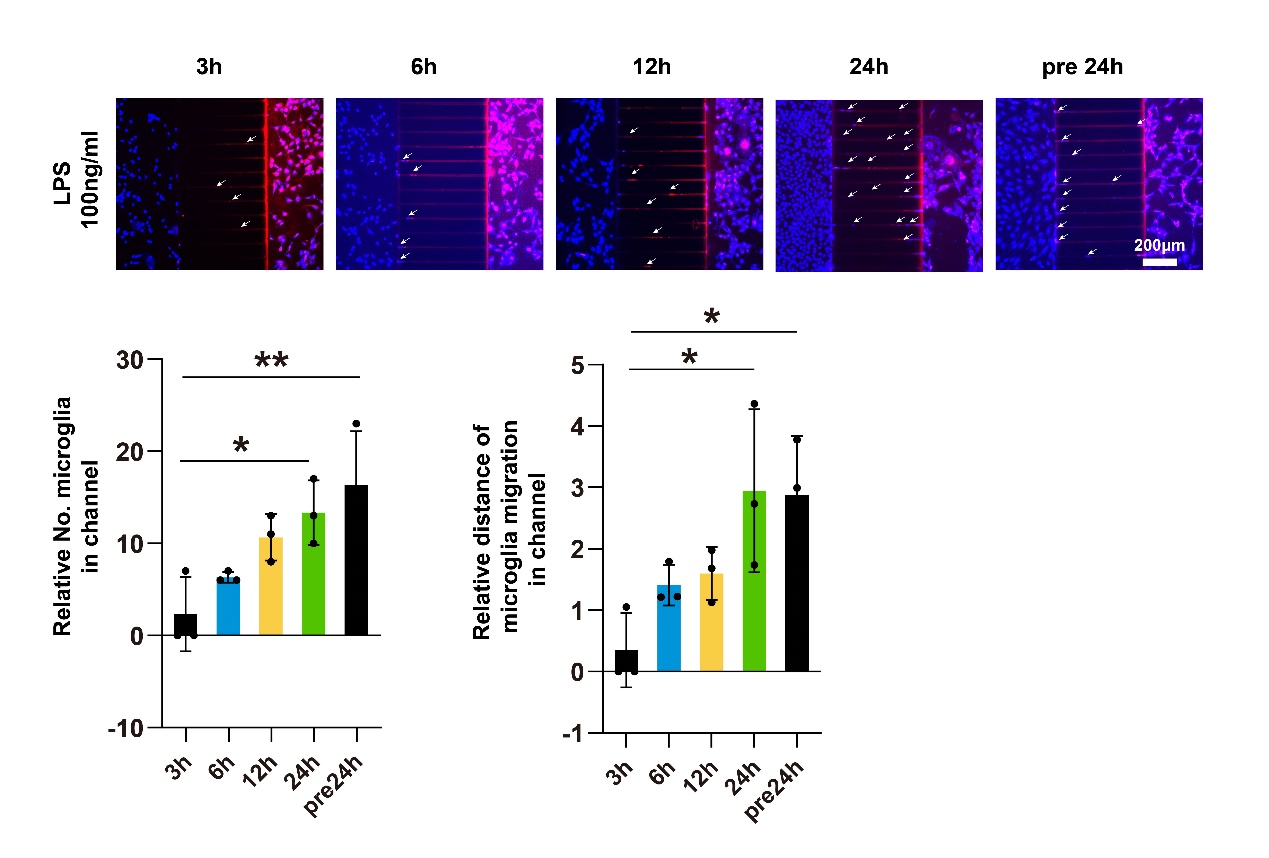


**Supplementary Fig. 3 |** **Recording of microglial responses following LPS treatment for human primary astrocytes (HA cells) at different time points in the microfluidic co-culture system.** Representative images of microglial cells labeled with cell tracker in side chamber and microgrooves. Scale bar: 200μm. The relative number of microglia in microgrooves and the relative distance of microglia migration in microgrooves are analyzed. Data are mean ± SD. ^*^p < 0.05; ^**^p < 0.01.


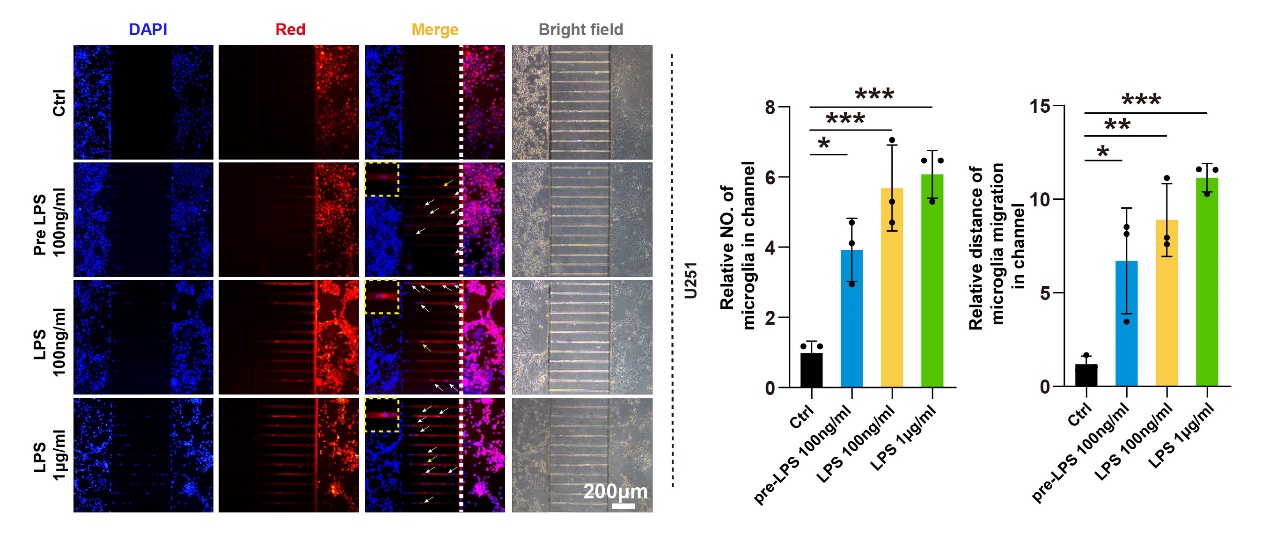
**Supplementary Fig. 4 | Recording of microglial responses following LPS treatment for U251 cells in the microfluidic co-culture system.** U251 cells (Procell, #U251) were plated in the central chamber and induced by LPS. Representative images of microglial cells labeled with cell tracker (red) in the side chamber. Scale bar: 200μm. The relative number of microglia in microgrooves and the relative distance of microglia migration in microgrooves are analyzed. 3 individual chips for each group. Data are mean ± SD. ^*^p < 0.05; ^**^p < 0.01; ^***^p < 0.001.


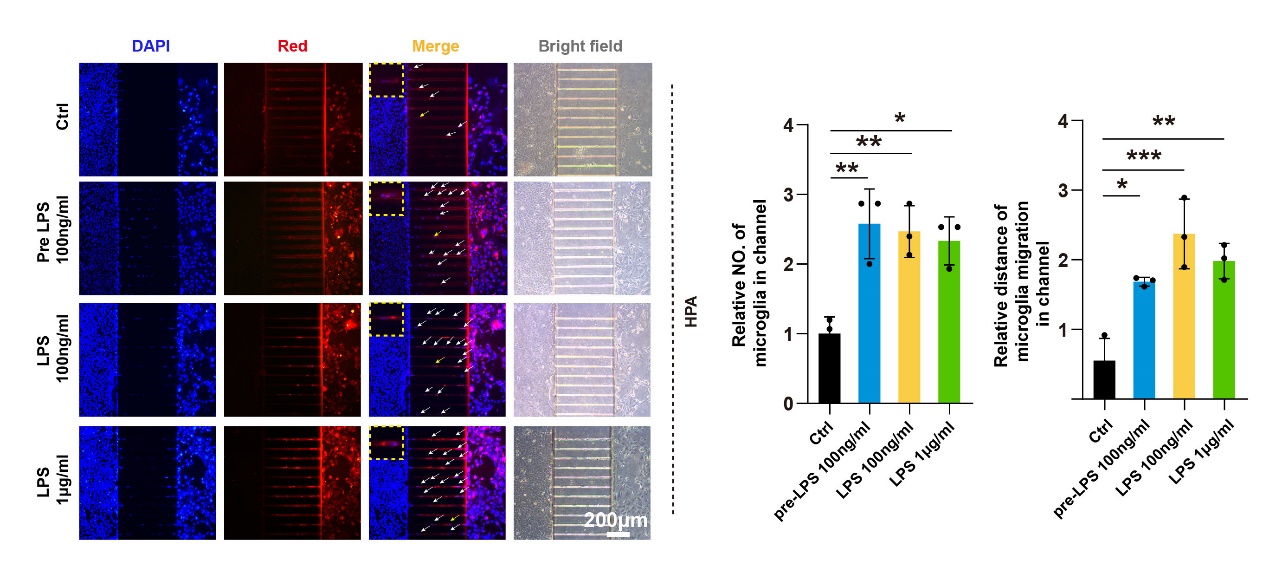
**Supplementary Fig. 5 | Recording of microglial responses following LPS treatment for human primary astrocytes (HPA cells) in the microfluidic co-culture system.** Astrocytes (human primary astrocytes, HPA, Pri-iCell-007, iCell corporation) were plated in the central chamber and induced by LPS. Representative images of microglial cells labeled with cell tracker (red) in the side chamber. Scale bar: 200μm. The relative number of microglia in microgrooves and the relative distance of microglia migration in microgrooves are analyzed. 3 individual chips for each group. Data are mean ± SD. ^*^p < 0.05; ^**^p < 0.01; ^***^p < 0.001.

**
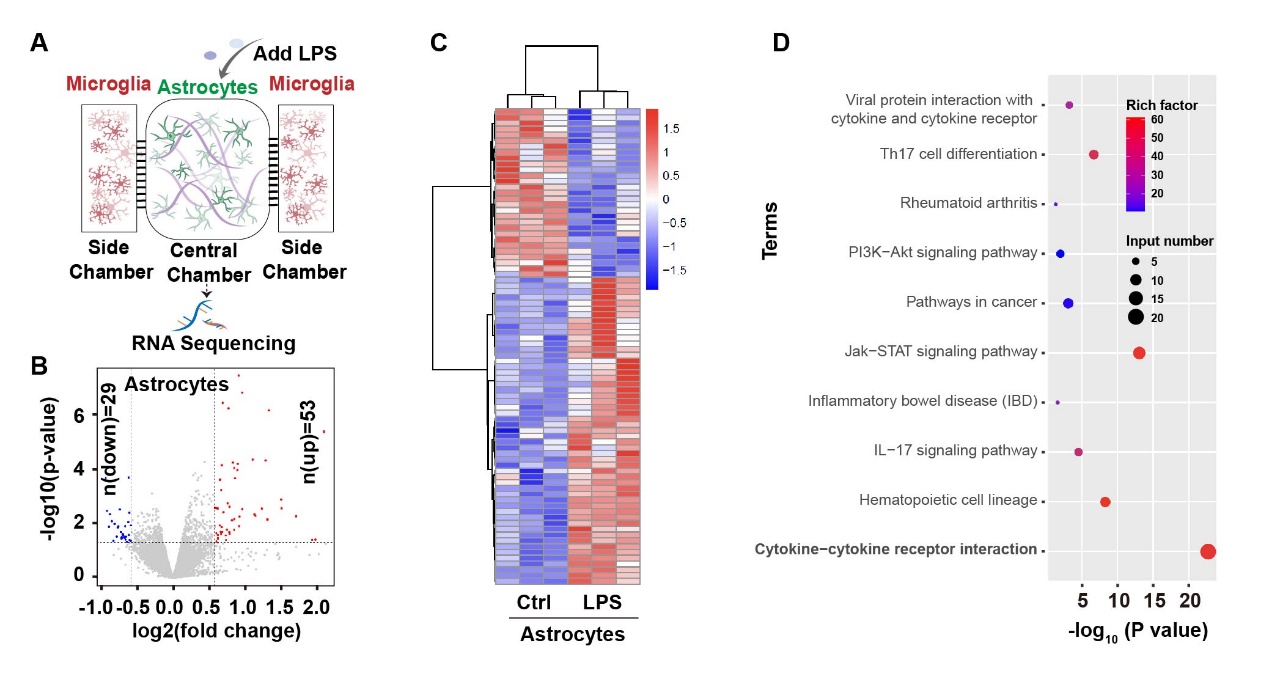
**

**Supplementary Fig. 6 | Transcriptional analysis of astrocytes responses to LPS in the microfluidic co-culture system. A**. Schematic description of human microfluidic co-culture system. **B**. Volcano plot of astrocytes gene expression in the central chamber. (p < 0.05, |fold change| > 1.5). **C**. Heat map showing transcriptional changes of astrocytes in the human microfluidic co-culture system. **D**. Pathway analysis, based on the KEGG database, highlights the enrichment of differentially expressed genes in central chamber astrocytes.


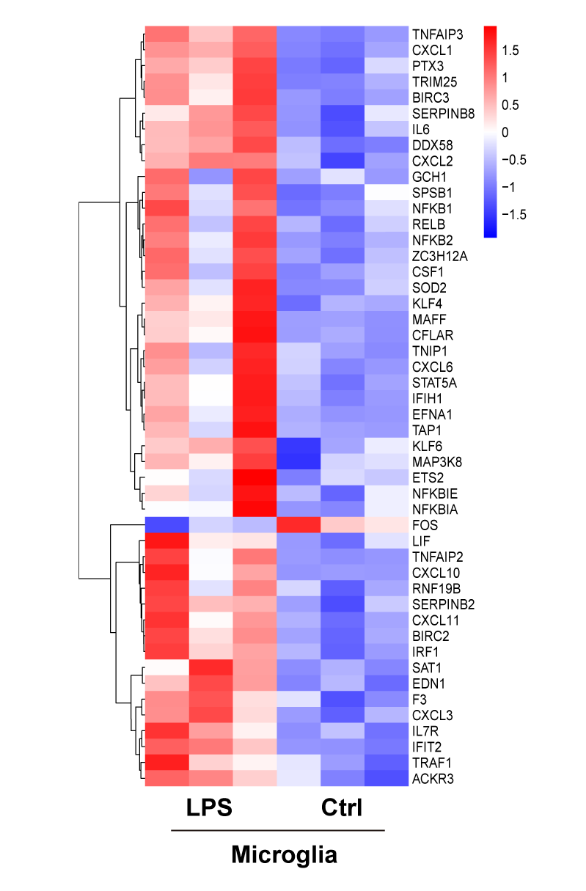
**Supplementary Fig. 7 | Transcriptional analysis of microglia responses to astrocyte activation in the microfluidic co-culture system.** Heat map showing expression of NF-κB signaling-related genes of microglia in the co-culture microfluidic system (n = 3).


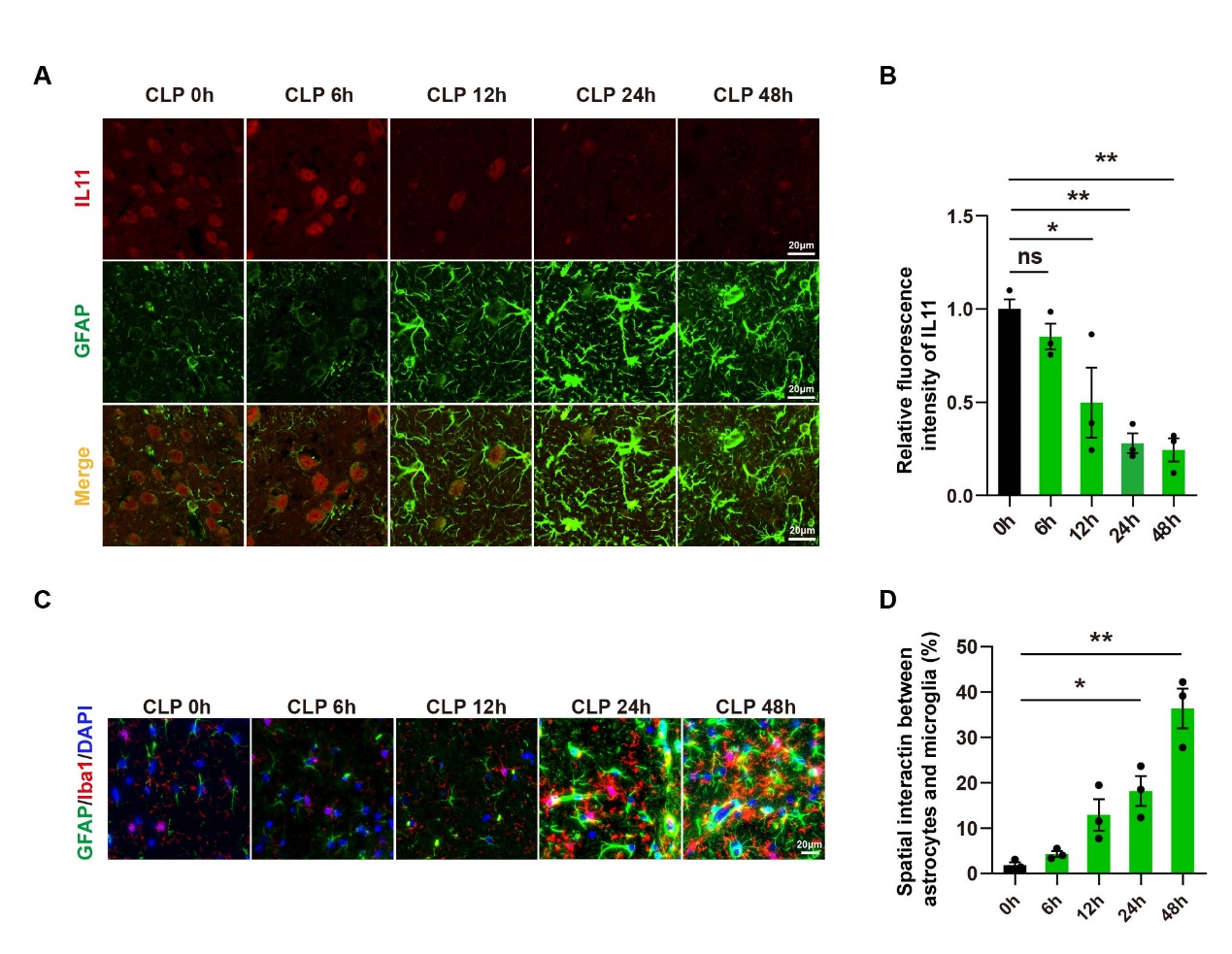

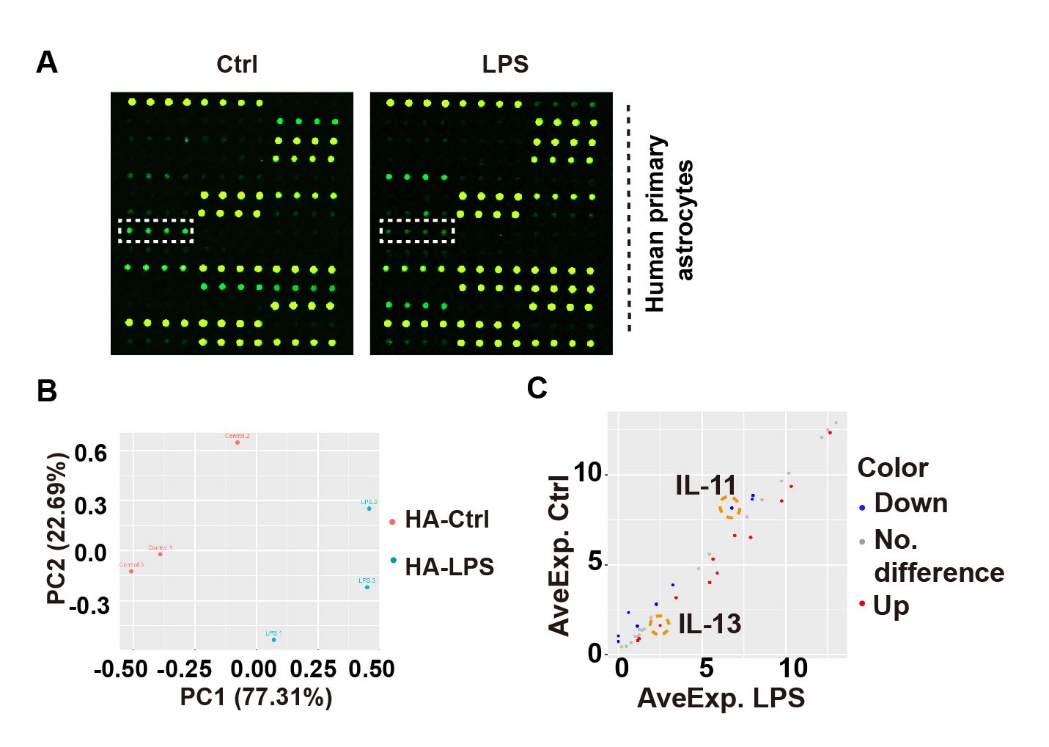
**Supplementary Fig. 8 | Cytokine profile. A.** Cytokine profile of HPA cells. Dots in the white boxes are references (IL11). **B.** The principle component analysis (PCA) are conducted on all differentially expressed proteins (DEPs) of HA cells between two groups. **C.** DEPs of HA cells are presented as Scatter plot.

**Supplementary Fig. 9 | Investigation of the temporal relationship between IL11 decrease and astrocytes/microglia interaction in the pathogenesis of SAE mouse models. A**. Immunofluorescent images showing brain slices immunostained for IL11 and GFAP at different time points post CLP (0h, 6h, 12h, 24h and 48h) (n=3). **B.** Quantification of IL11 immunofluorescent intensity based on (A). **C**. Immunofluorescent images showing brain slices immunostained for GFAP and Iba1 at different time points post CLP (0h, 6h, 12h, 24h and 48h) (n=3). **D**. Quantification of spatial interaction between astrocytes (GFAP) and microglia (Iba1) based on (C). Data are mean ± SD. ^*^p < 0.05; ^**^p < 0.01.


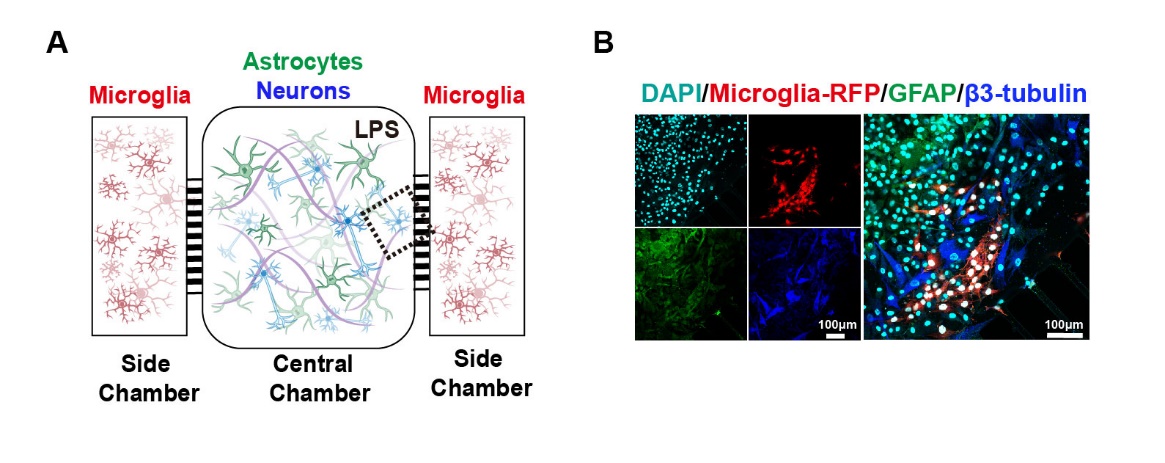
**Supplementary Fig. 10 | Neurons/Astrocytes/microglia tri-culture system. A**. Scheme of a microfluidic neurons/astrocytes/microglia tri-culture system. **B**. Immunofluorescent images showing human astrocytes and neurons cultured in the central chamber, and human microglia labeled with RFP cultured in side chambers. The area of the image was indicated in (A) with dashed black box.


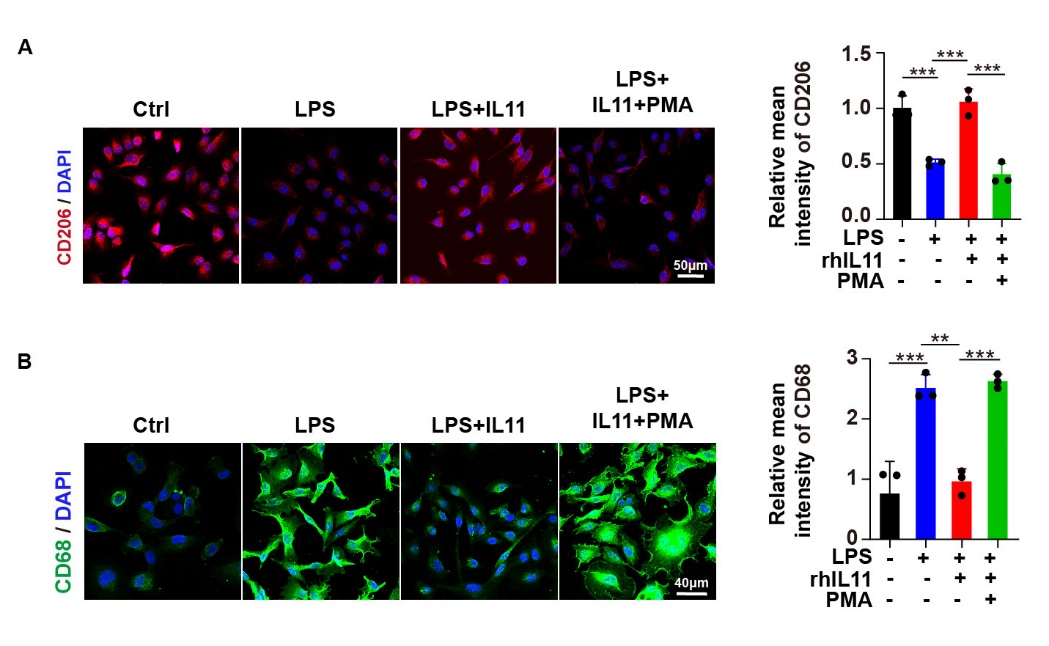
**Supplementary Fig. 11 | Testing the roles of IL11 in regulating M1/M2 microglial polarization. A. & B.** Immunofluorescent images showing microglia immunostained for CD206 (red) and CD68(green). The levels of expression of M2 cell marker (A) CD206 and M1 cell marker (B) CD68 were quantified based on immunofluorescence intensity. Scale bar: 50 μm, 40 μm. n = 3 ROIs from 3 individual chips for each group. Data are mean ± SD. ^**^p < 0.01; ^***^p < 0.001.


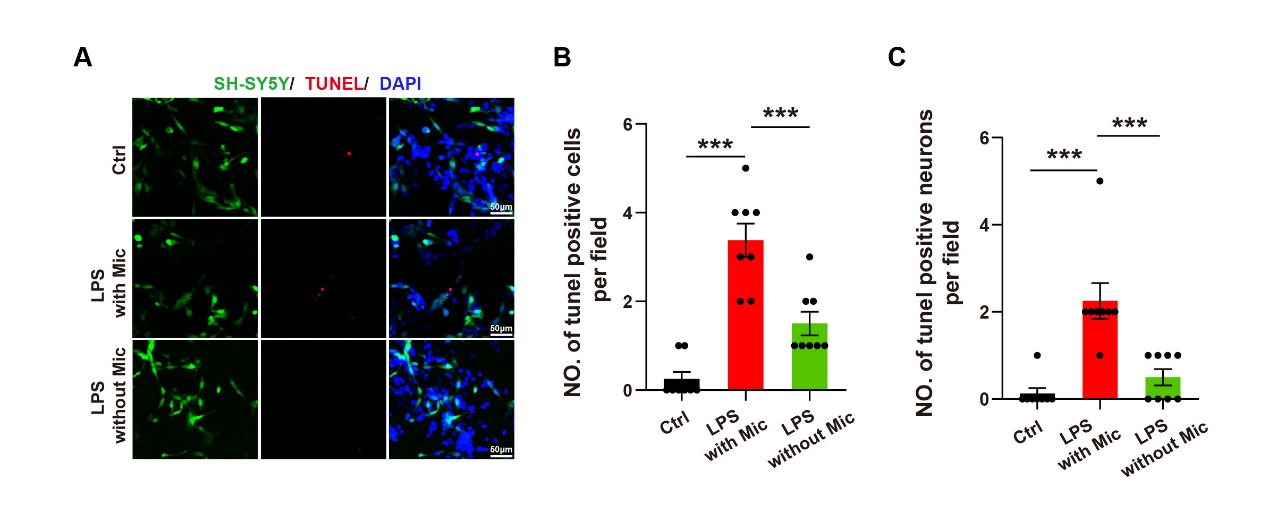
**Supplementary Fig. 12 | An ablation study was performed to test the roles of microglia in the pathogenesis of SAE. A**. Fluorescent images showing TUNEL positive cells (cell apoptosis) in central neurons/astrocytes compartment following LPS treatment with or without microglia. SH-SY5Y cells were labelled with GFP. **B**. Quantification of TUNEL positive cells based on (A). **C**. Quantification of TUNEL positive SH-SY5Y cells based on (A). Two random fields were analyzed for each chip. Mic: microglia. n = 8 ROIs from ≥3 individual chips for each group Data are mean ± SD. ^***^p < 0.001.

**Supplementary Table 1 | Primer sequences used for qRT-qPCR analysis in this study.**

| Gene | Forward primer (5’-3’) | Reverse primer (5’-3’) |
| --- | --- | --- |
| IL11 | GAC ATG AAA CAG CAG GCT AC | CAC CCA CAA TCC CAC CTC |
| IL11Rα | CTG GGC TAG GGC ATG AAC TG | CTG GGA CTC CAA GTG CAA GA |
| gp130 | CGG ACA GCT TGA ACA GAA TGT | ACC ATC CCA CTC ACA CCT CA |
| GAPDH | CTTCACGACCATGGAGAAGG | CCAAGCAGTTGGTGGTGCAG |

**Supplementary Table 2 | Primary antibodies used for immunofluorescence in this study.**

| **Antibody** | **Vendor** | **Catalog#** | **Dilution** |
| --- | --- | --- | --- |
| Anti-GFAP | Cell Signaling Technology | 3670S | 1:100 |
| Anti-GFAP | Proteintech Group | 16825-1-AP | 1:100 |
| Anti-S100β | Proteintech Group | 15146-1-AP | 1:100 |
| Anti-IBA1 | Abcam | ab178847 | 1:100 |
| Anti-IBA1 | Proteintech Group | 66827-1-Ig | 1:100 |
| Anti-CD11b | Abcam | ab52478 | 1:100 |
| Anti-IBA1 | Wako | 019-19741 | 1:500 |
| Anti-AQP4 | Proteintech Group | 16473-1-AP 1 | 1:200 |
| Anti-IL11 | Proteintech Group | 55169-1-AP | 1:200 |
| Anti-CD68 | Cell Signaling Technology | 76437S | 1:200 |
| Anti-CD206 | Abcam | Ab64693 | 1:500 |
| Anti-CD68 | Proteintech Group | 66231-2-1g | 1:100 |
| Anti-NFkB1 | Proteintech Group | 66992-1-Ig | 1:200 |
| Anti-MAP2 | Sigma | M1406 | 1:200 |

**Supplementary Table 3 | Primary antibodies used for Western blot in this study.**

| **Antibody** | **Vendor** | **Catalog#** | **Dilution** |
| --- | --- | --- | --- |
| Anti-GFAP | Millipore | MAB360 | 1:1000 |
| Anti-S100β | Proteintech Group | 15146-1-AP | 1:1000 |
| Anti-IBA1 | Abcam | ab178847 | 1:1000 |
| Anti-IBA1 | Proteintech Group | 66827-1-Ig | 1:1000 |
| Anti-AQP4 | Proteintech Group | 16473-1-AP 1 | 1:1000 |
| Anti-β-actin | Cell Signaling Technology | 3700s | 1:2000 |
| Anti-GAPDH | Abcam | ab9484 | 1:1000 |
| Anti-p-P65 | Wanleibio | WL02169 | 1:500 |
| Anti-P-NFkB1 | Bioss | bs-5512R | 1:500 |
| Anti-P-IkBα | Bioss | bs-5515R | 1:500 |
| Anti-IKKα/β | Wanleibio | WL01900 | 1:500 |
